# Supplementary material for: Shifts in naturalistic behaviors induced by early social isolation stress are associated with adult binge-like eating in female rats
Source: Front Behav Neurosci. 2024 Dec 12;18:1519558. doi: 10.3389/fnbeh.2024.1519558 (PMC11669510; doi:10.3389/fnbeh.2024.1519558)
Supplement: Supplementary file 1 [file Table_1.DOCX]

***Supplementary Material***

1. **Supplementary Figures**


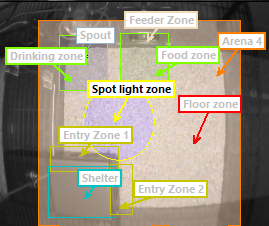

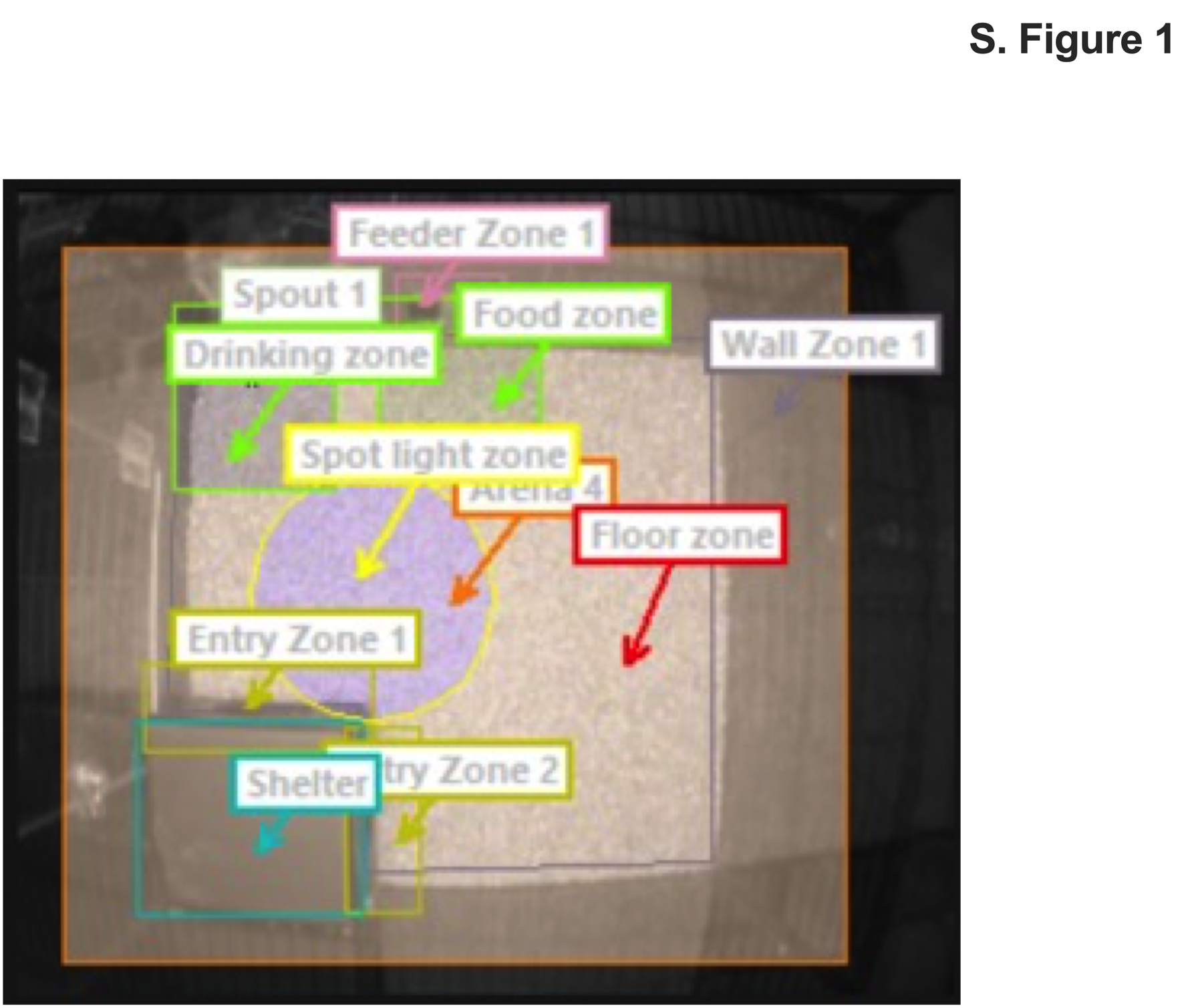
**S. Fig. 1.1** Aversive spotlight location and zones within the observational home cage.


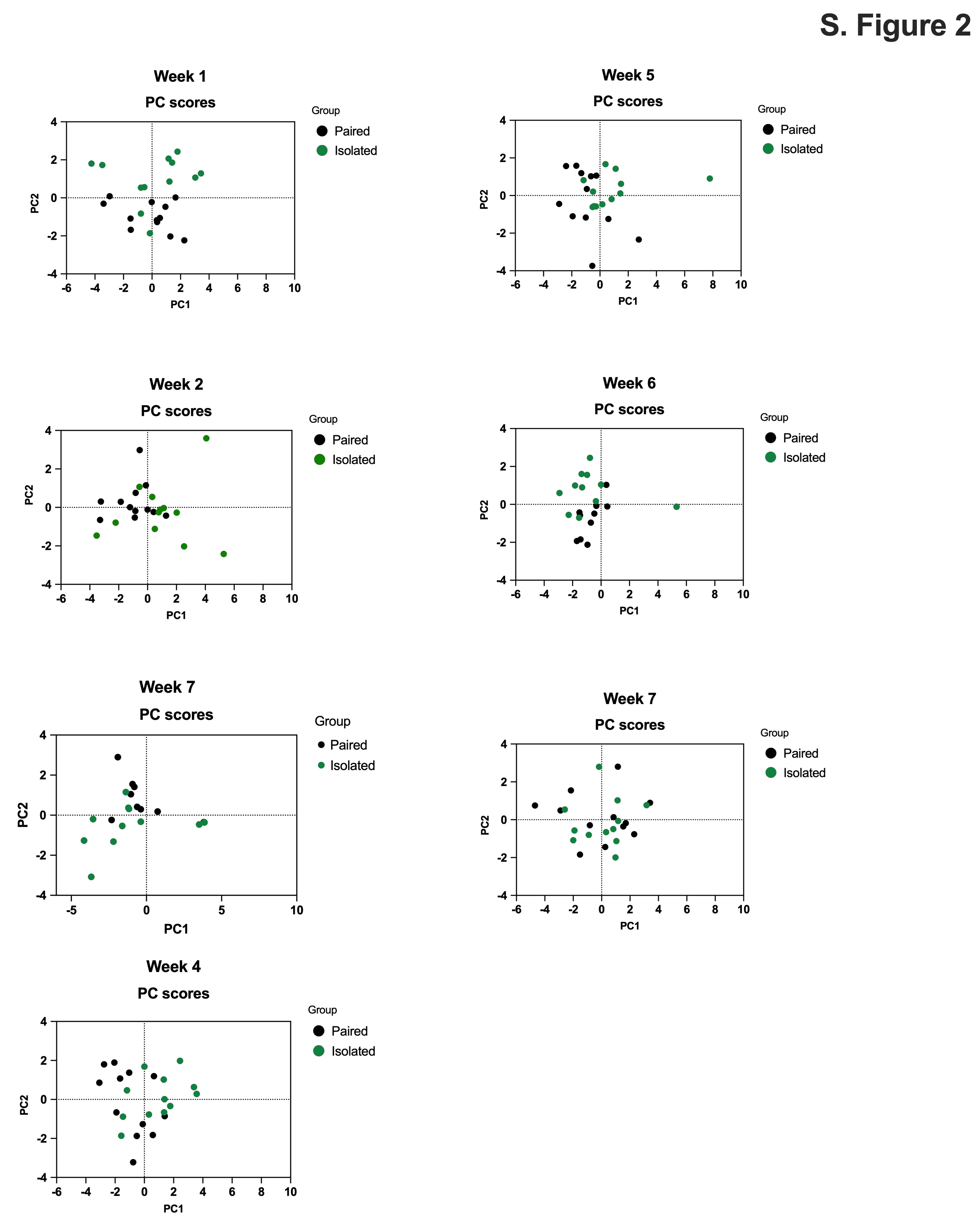
**S. Fig. 2.** PCA of 10 naturalistic behaviors distinguishes Isolated and Paired animals. The Paired animals in black, Isolated in green for all seven weeks. All the behaviors were standardized. Principal components were selected based off greater than 75% total explained variation.


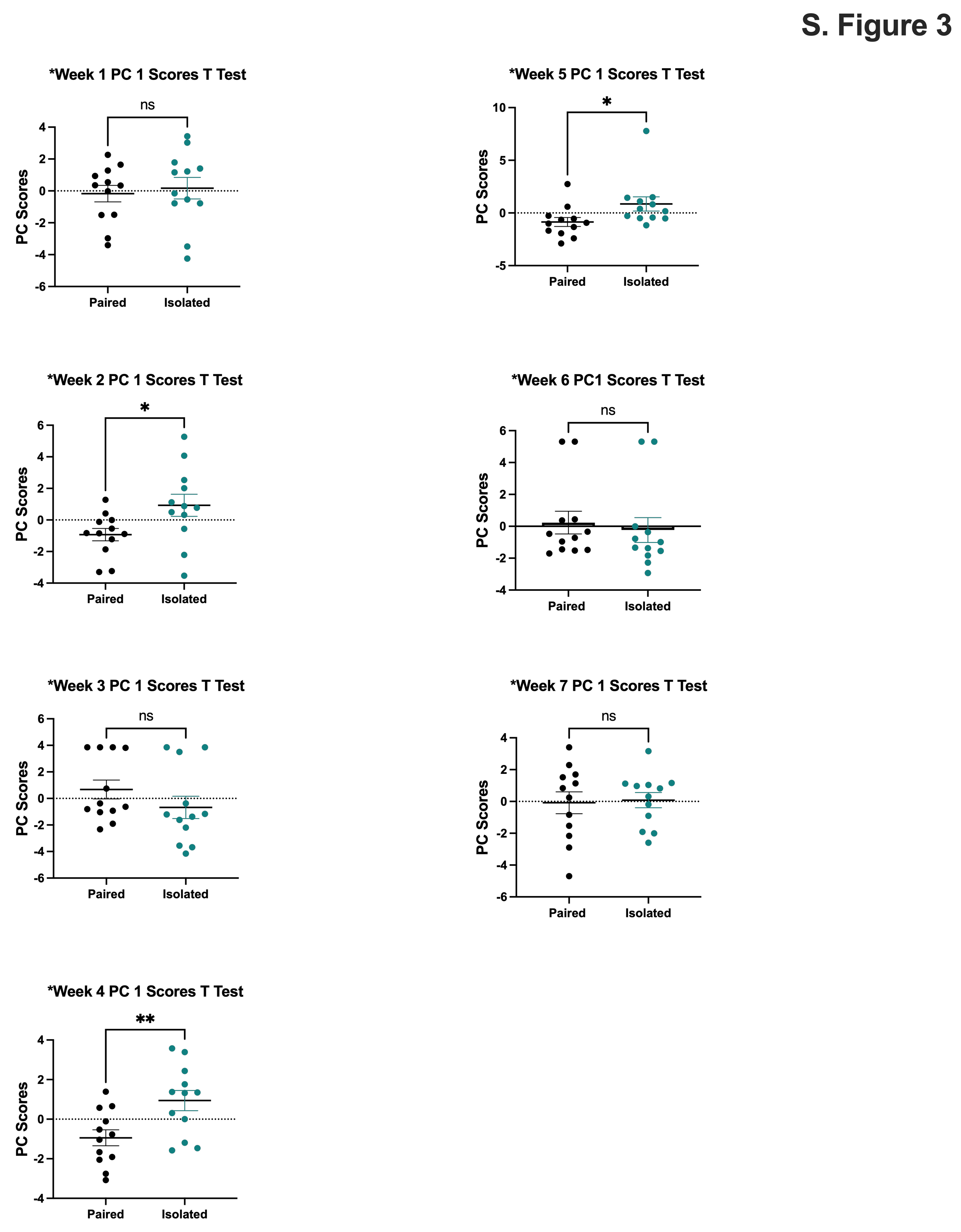
**S. Fig. 3.** PC1 scores are altered in Isolated and Paired animals. The Paired animals in black, Isolated in green for all seven weeks. Paired and Isolated, n=12 per group. Data presented with SEM. * p < 0.05, ** p < 0.01, *** p < 0.001, **** p <0.0001; Student t- tests.


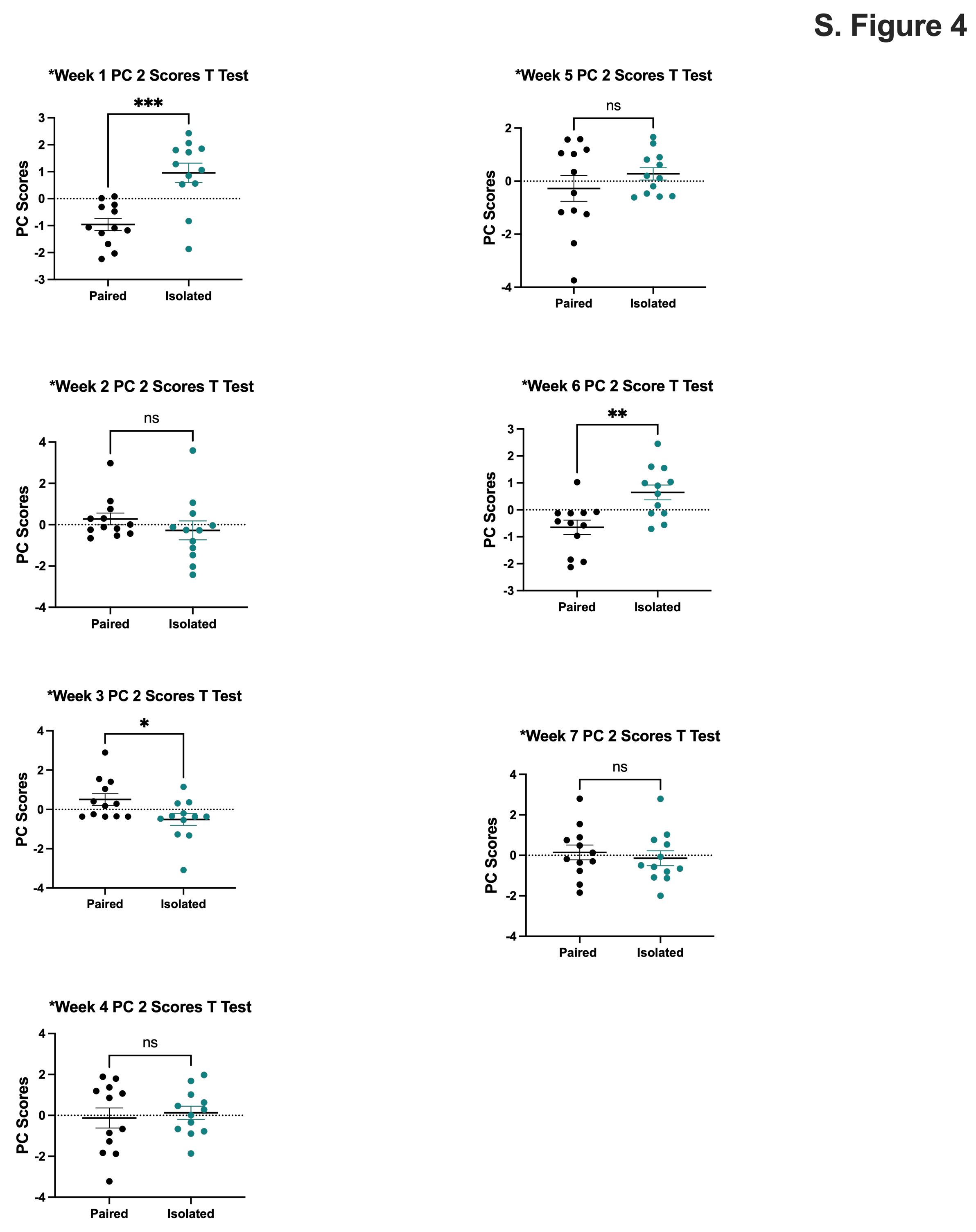
**S. Fig. 4.** PC2 scores are altered in Isolated and Paired animals. The Paired animals in black, Isolated in green for all seven weeks. Paired and Isolated, n=12 per group. Data presented with SEM. * p < 0.05, ** p < 0.01, *** p < 0.001, **** p <0.0001; Student t- tests.


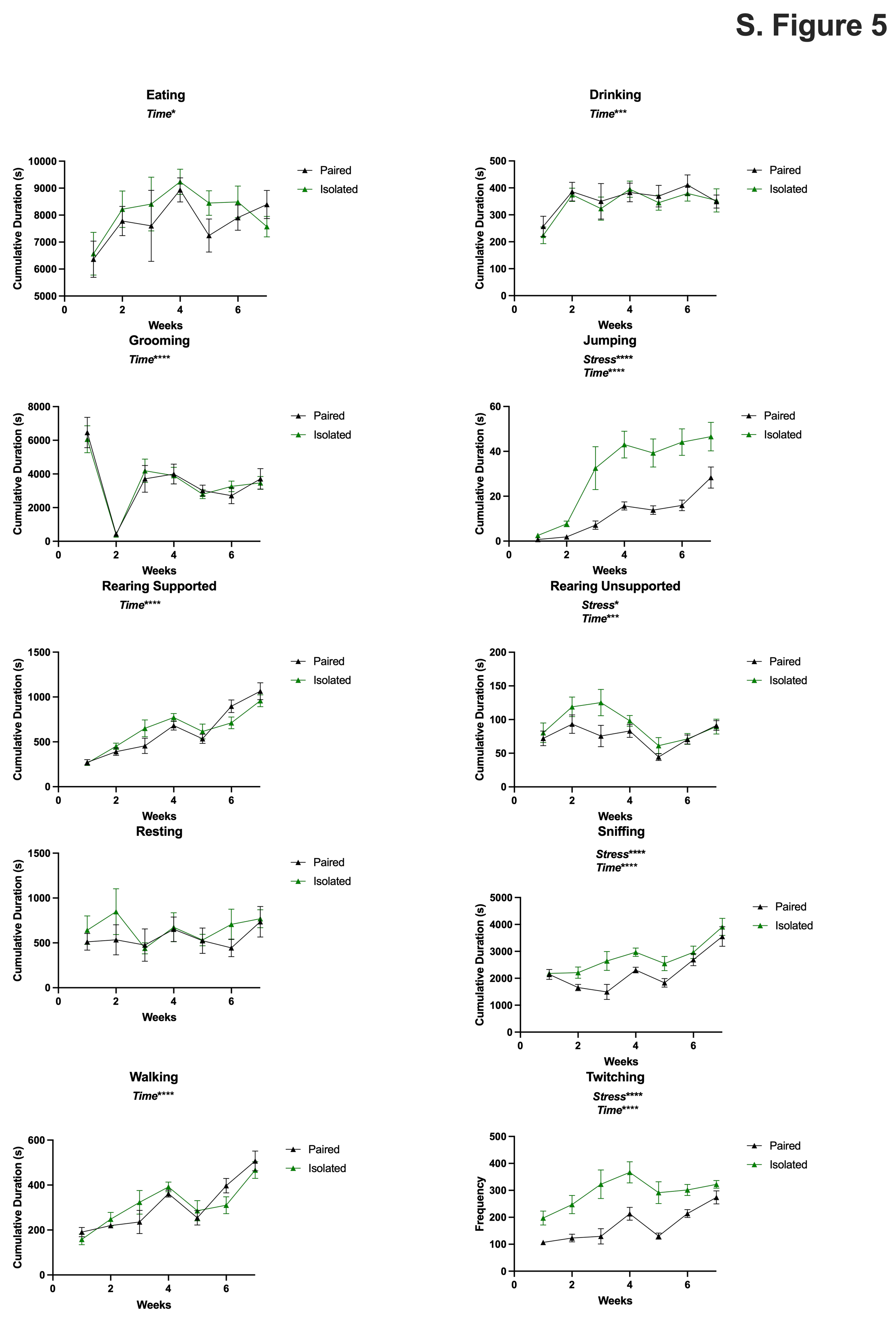
**S. Fig. 5.** Weekly trajectories for each naturalistic behavior for all seven weeks. Paired are in black, Isolation in green. Data presented with SEM. Paired and Isolated, n=12 per group. * p < 0.05, ** p < 0.01, *** p < 0.001, **** p <0.0001; Two-Way ANOVA.

**S. Fig. 6.** Shifted behaviors capture the phenotypic profile of SI animals. **(A)** Shifted behaviors include Rearing Unsupported, Jumping, Sniffing, and Twitching. **(B)** Nonshifted included the remaining six behaviors. Paired and Isolated, n=12. Data presented with SEM. * p < 0.05, ** p < 0.01, *** p < 0.001, **** p <0.0001; Student t- tests.


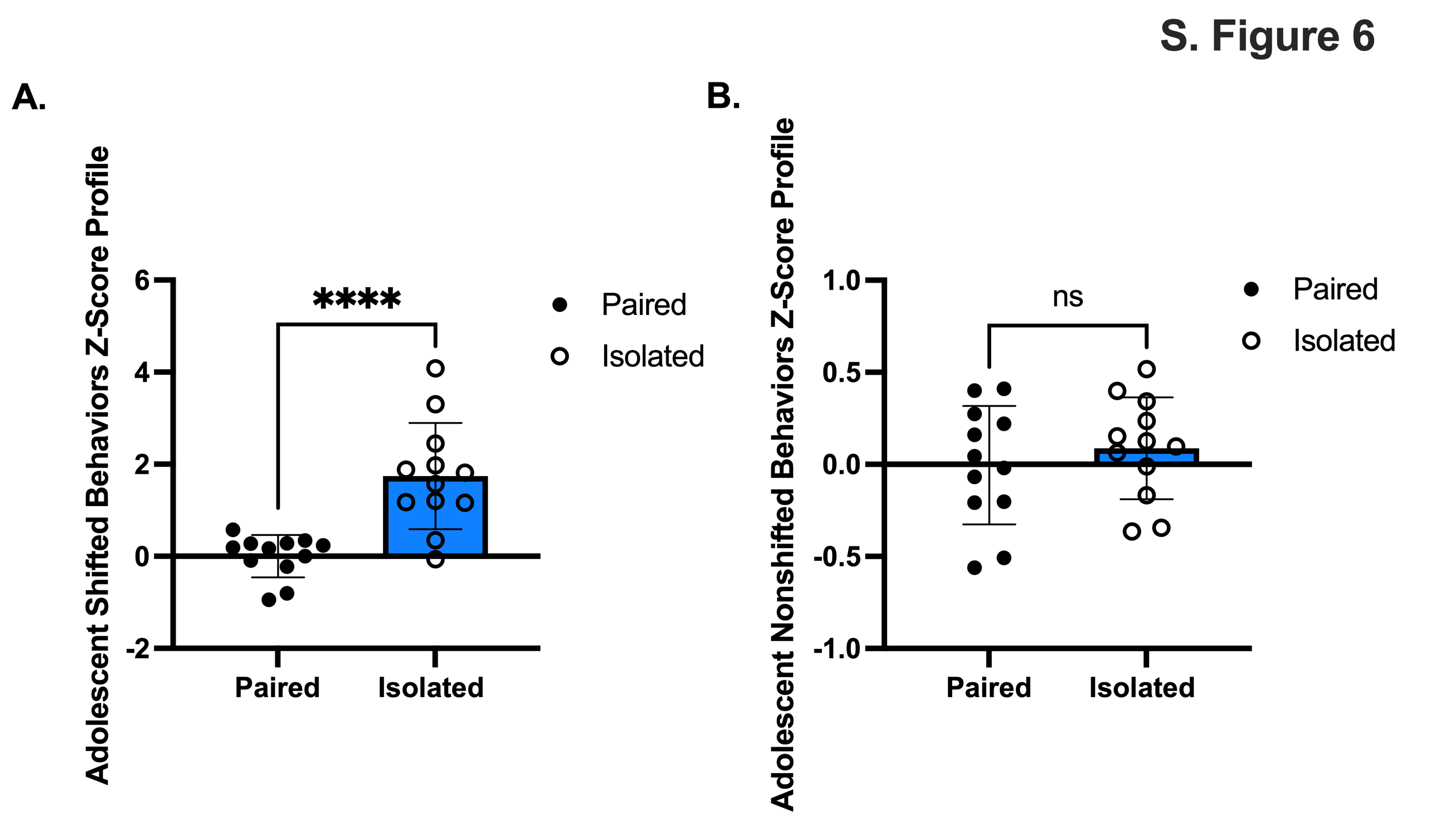


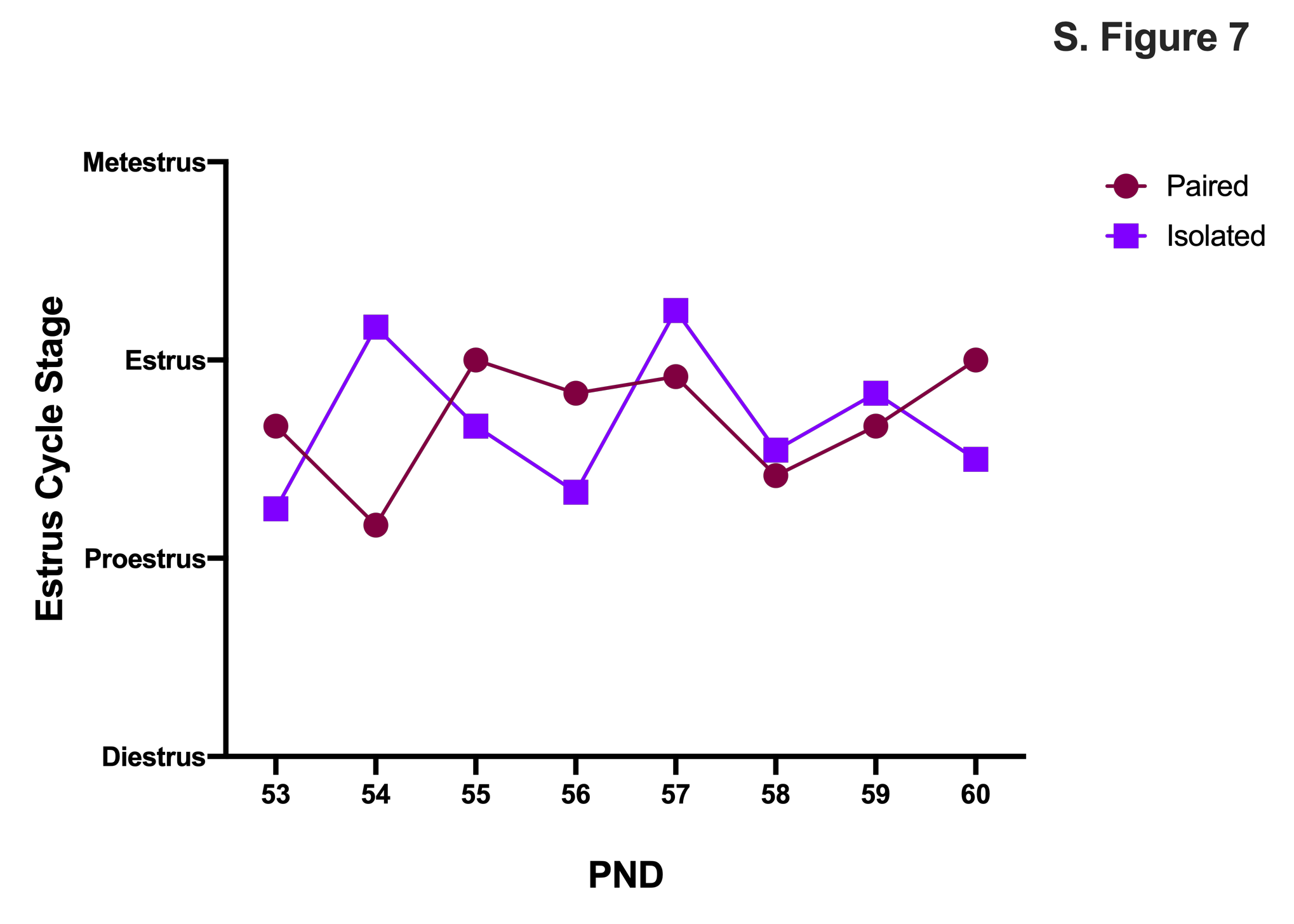
**S. Fig. 7.** Estrus Cyclicity does not differ between Paired and Isolated groups during adolescence.

**S. Fig. 8.** An aversive spotlight challenge. The dark grey rectangle depicts the start and stop of the spotlight hour. Each behavior is shown for all 7 weeks. Paired are in black, Isolation in green. Data presented with SEM. Paired and Isolated, n=12 per group. * p < 0.05, ** p < 0.01, *** p < 0.001, **** p <0.0001; Two-Way ANOVA.


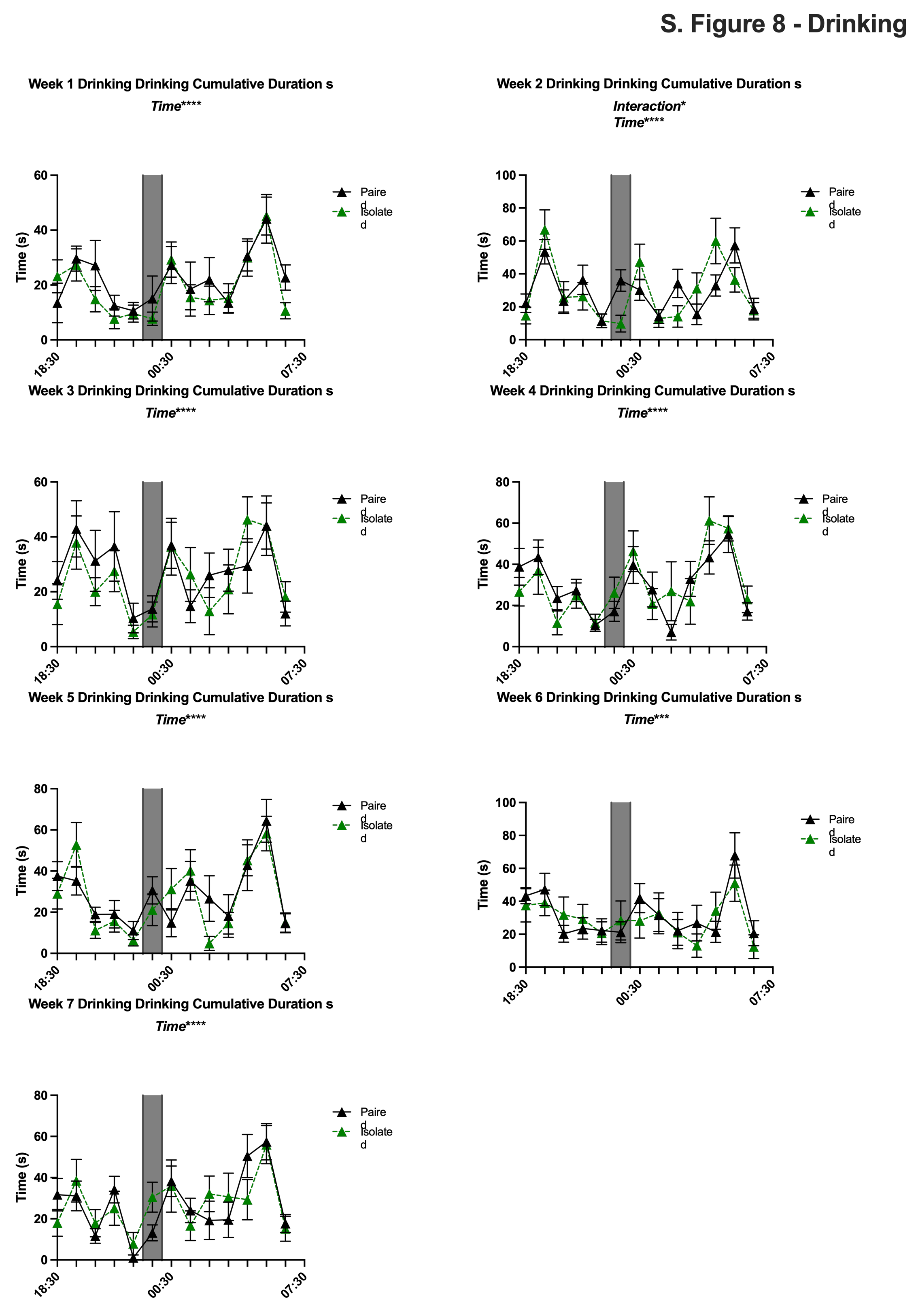

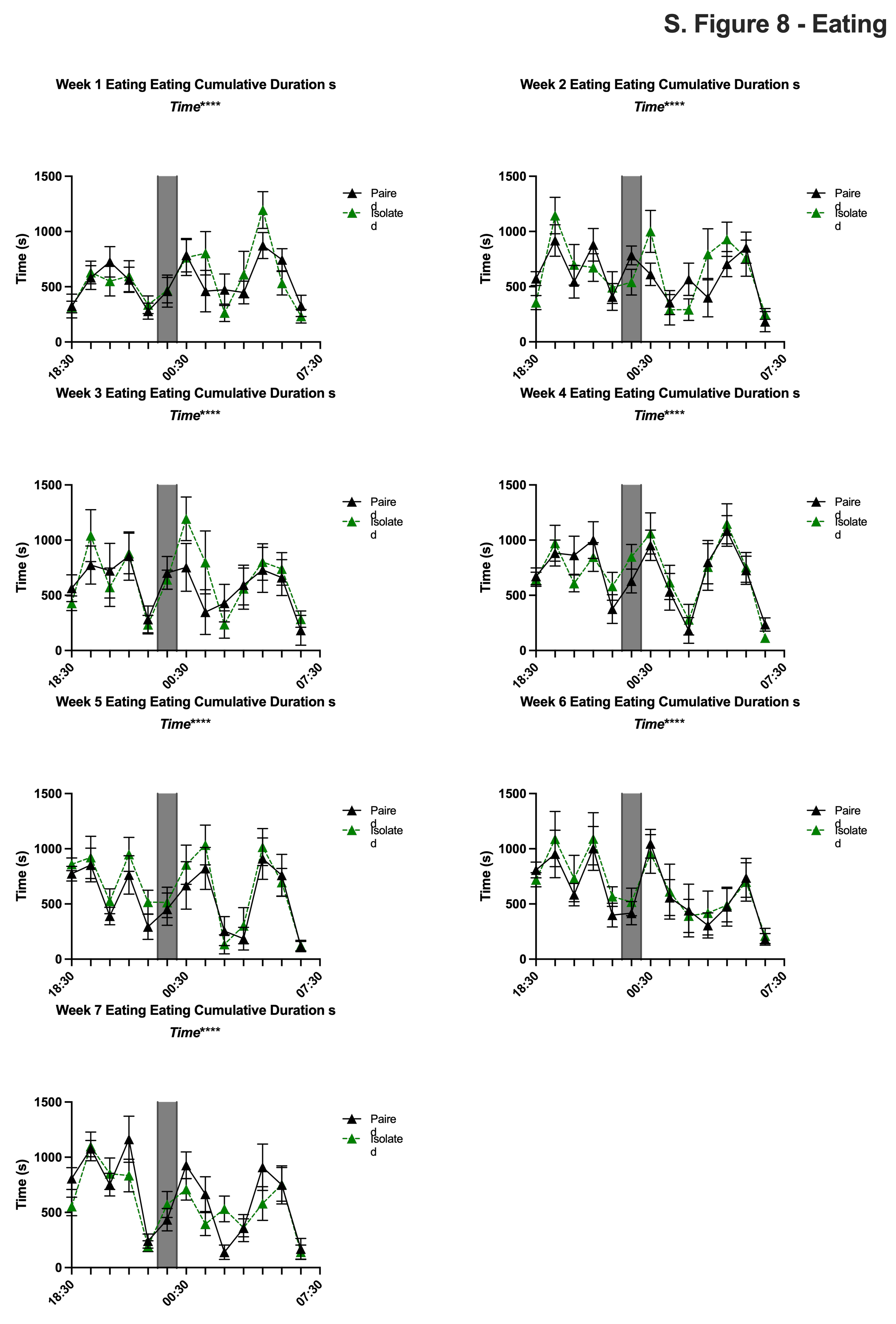

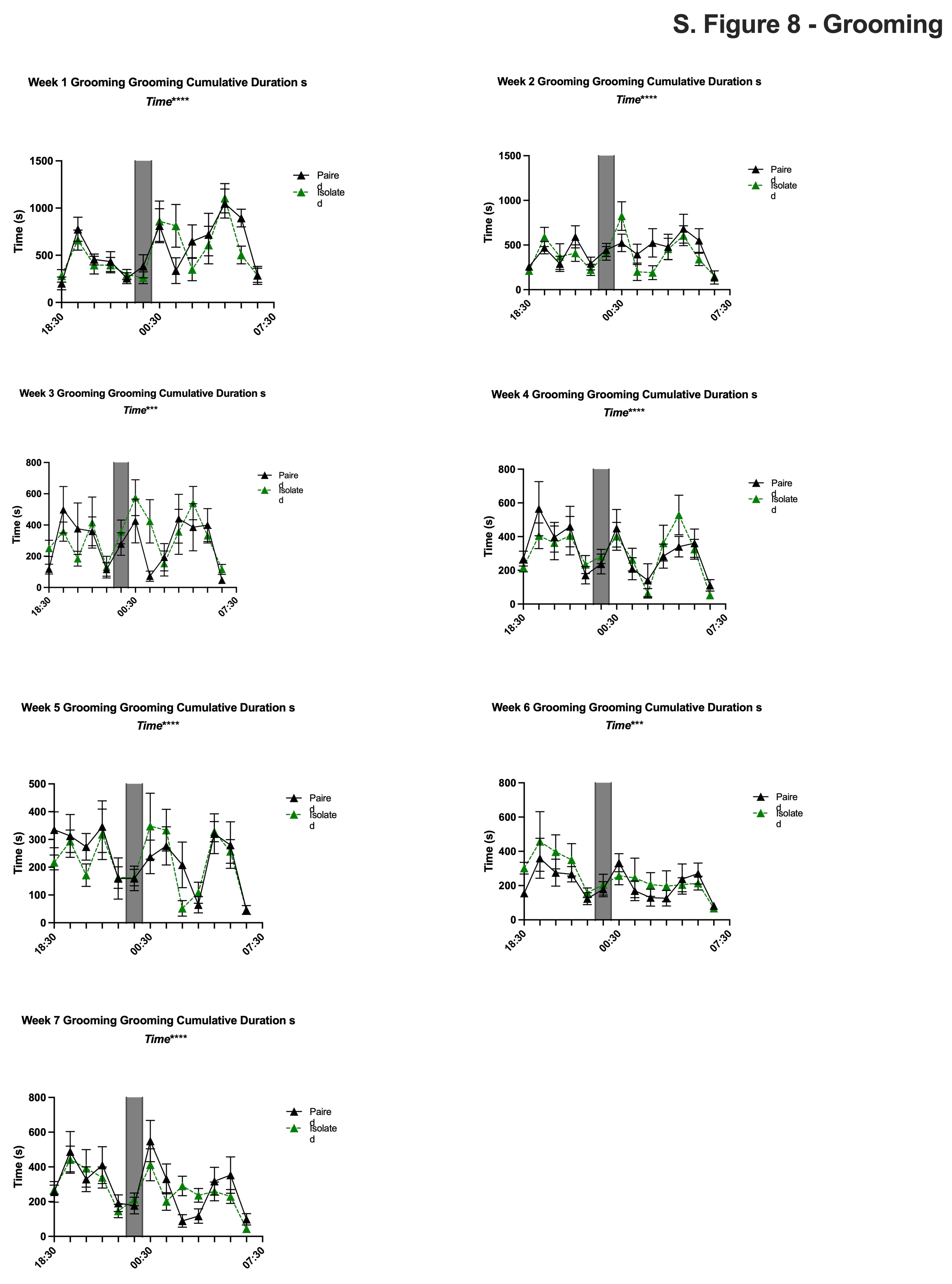

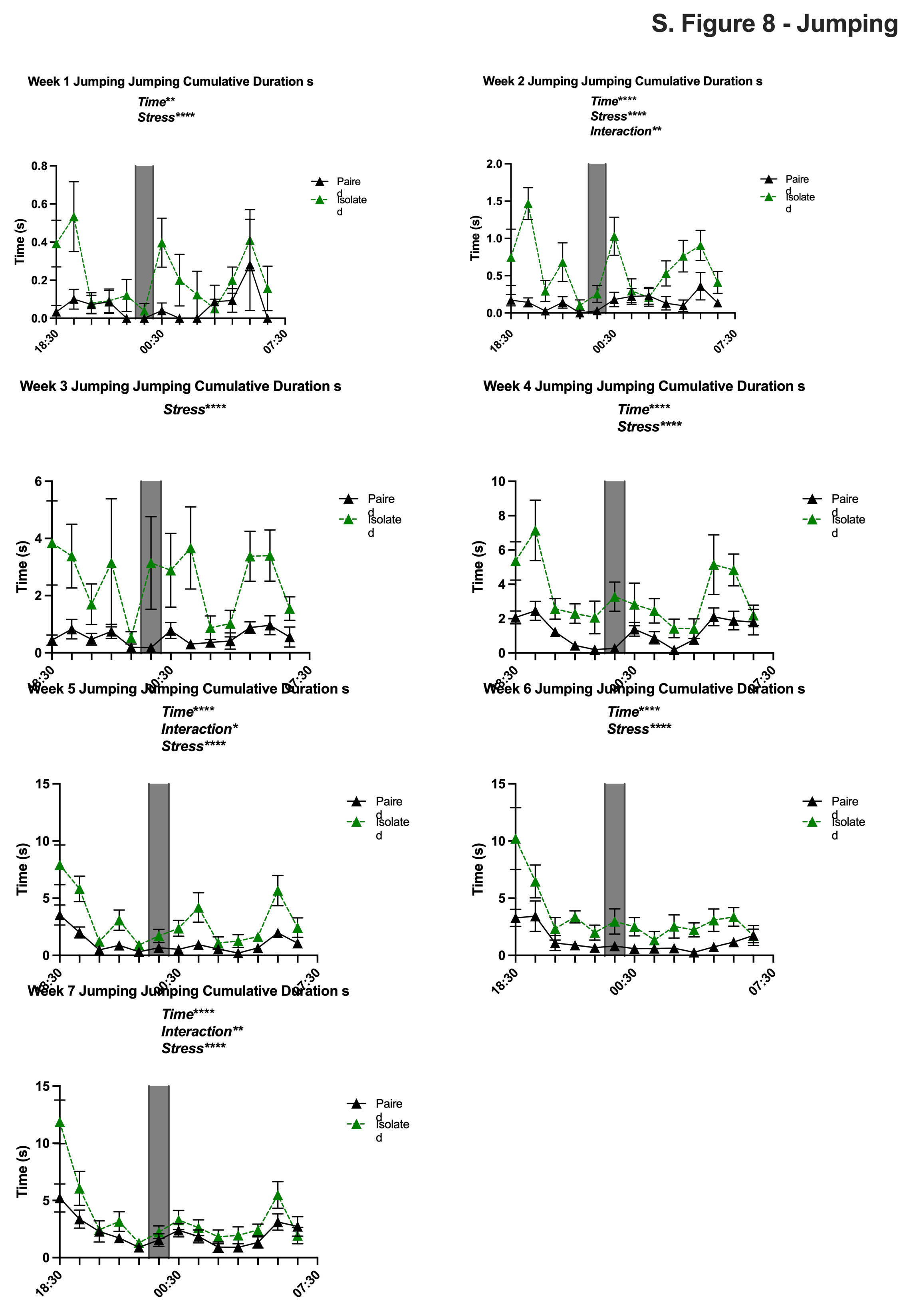

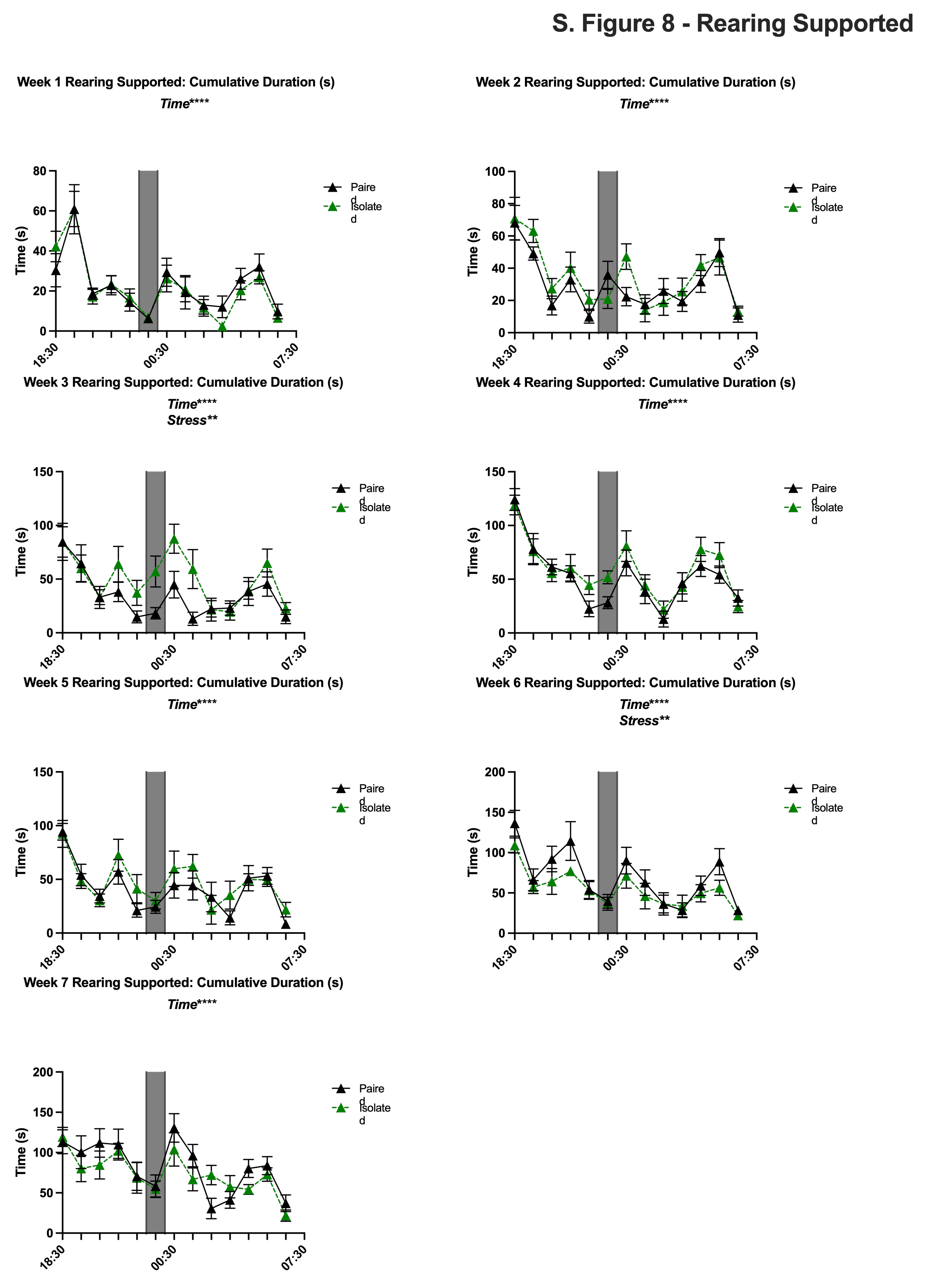

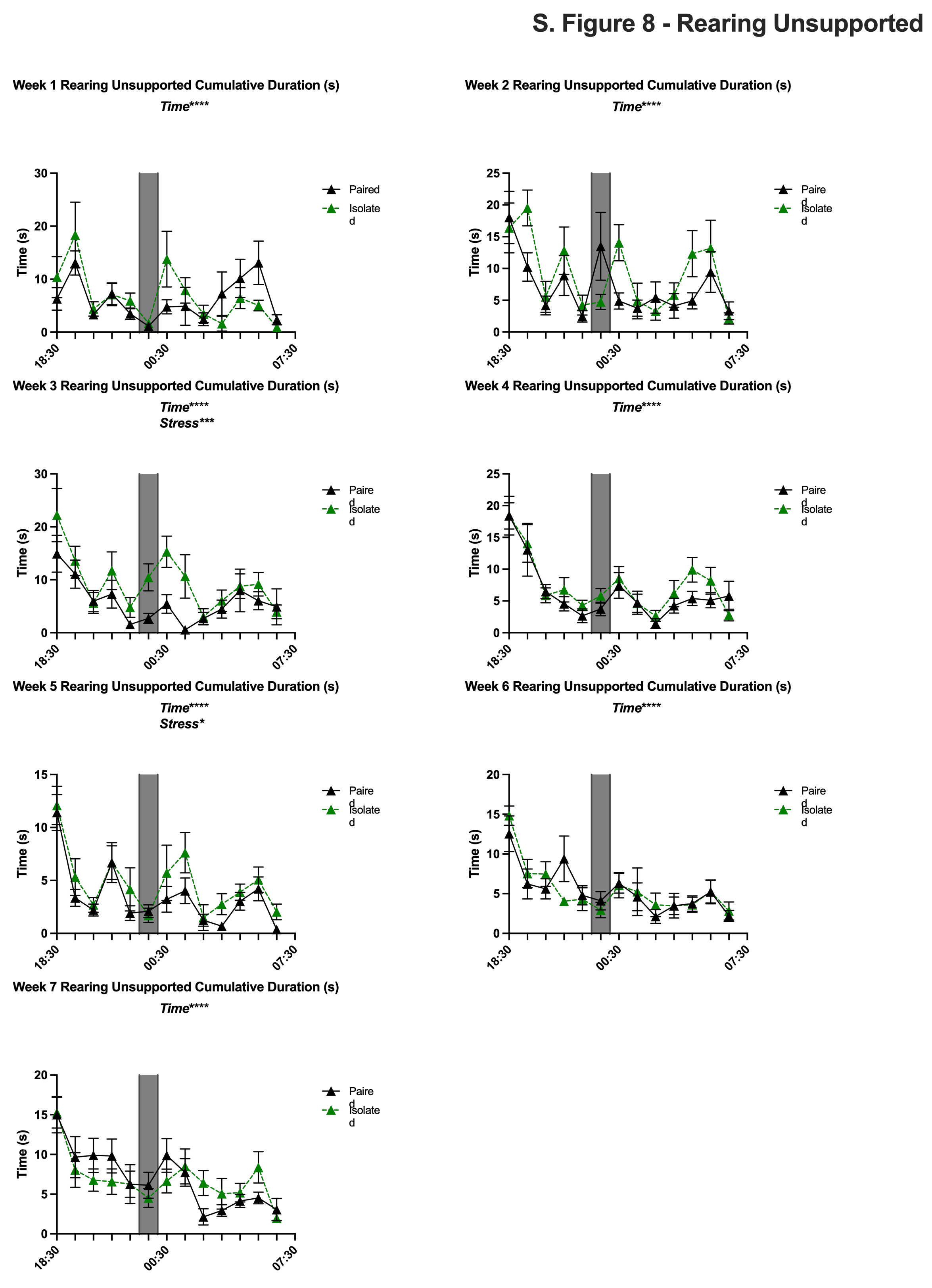

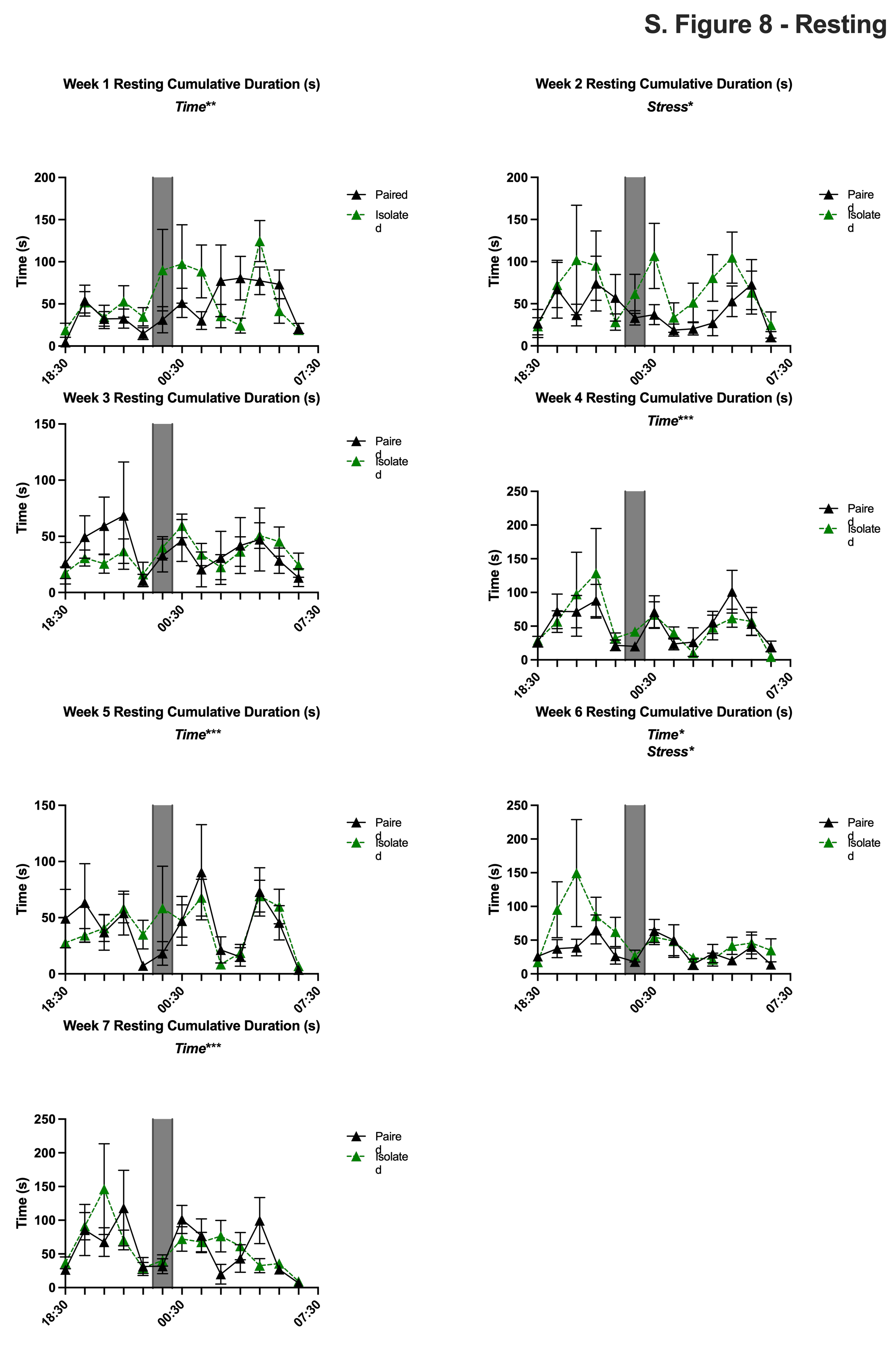

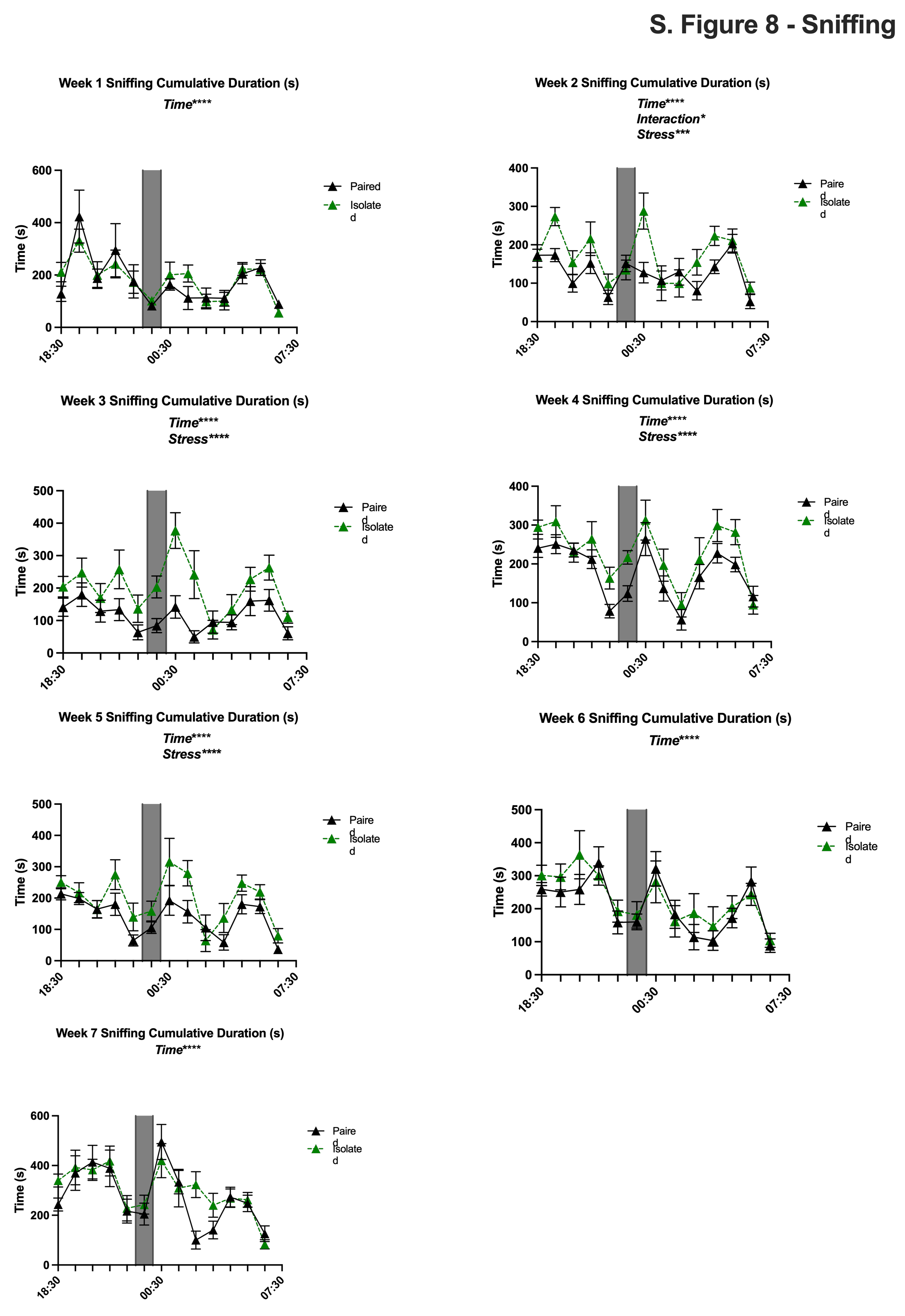


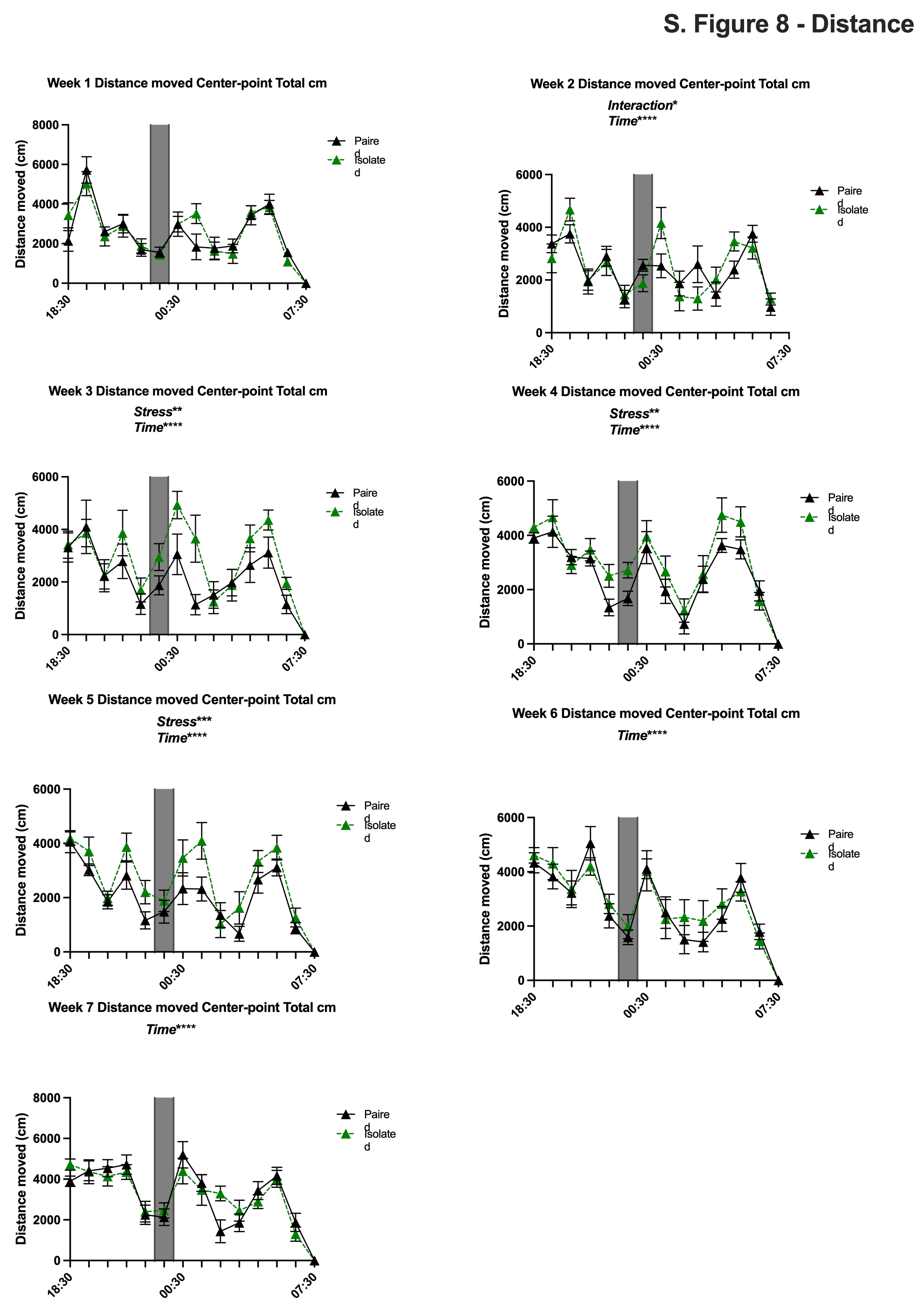


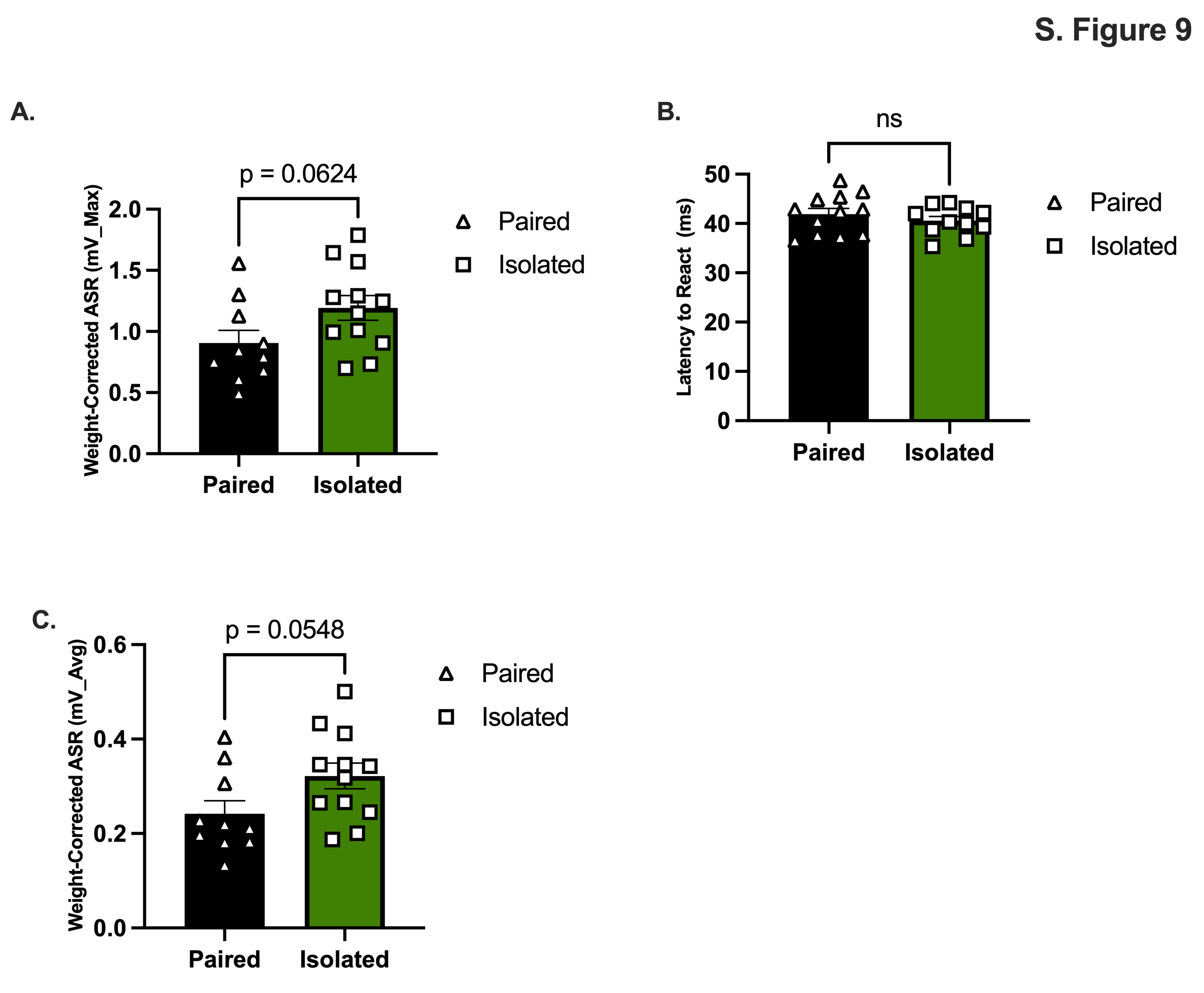
**S. Fig. 9.** Acoustic startle Reactivity. Isolated animals hyper react compared to Paired counterparts. **(A)** Weight-corrected maximum amplitude. **(B)** Latency to react in milliseconds. **(C)** Weight-corrected average amplitude. Data presented with SEM. * p < 0.05, ** p < 0.01, *** p < 0.001, **** p <0.0001; Student t- tests for parametric and Mann-Whitney for nonparametric.


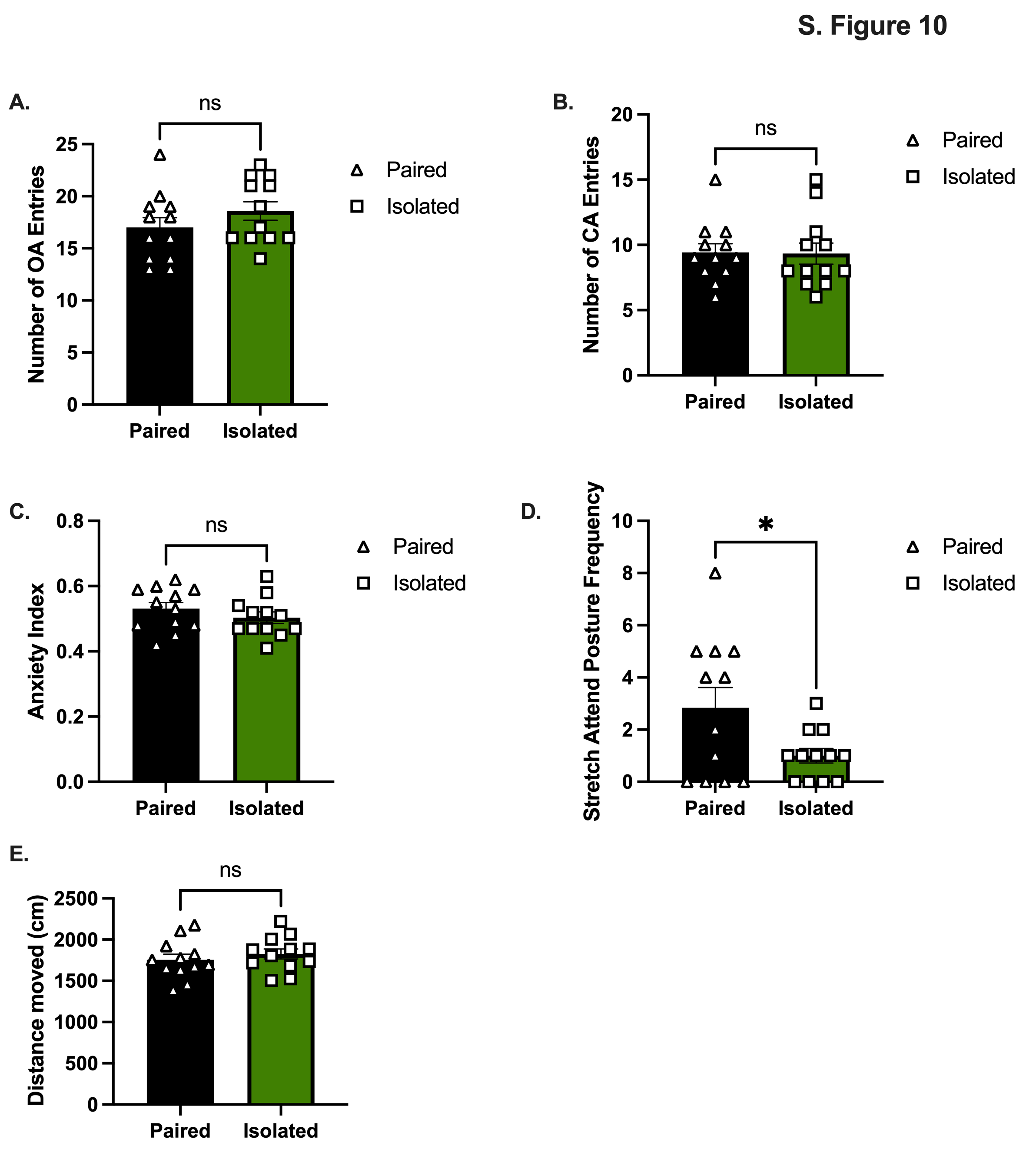
**S. Fig. 10.** Elevated Plus Maze. **(A, B, C, and E)** No differences in EPM. **(D)** Stretch attend posture is significantly decreased in Isolated animals. OA = open arms; CA = closed arms. Data presented with SEM. * p < 0.05, ** p < 0.01, *** p < 0.001, **** p <0.0001; Student t- tests for parametric and Mann-Whitney for nonparametric.


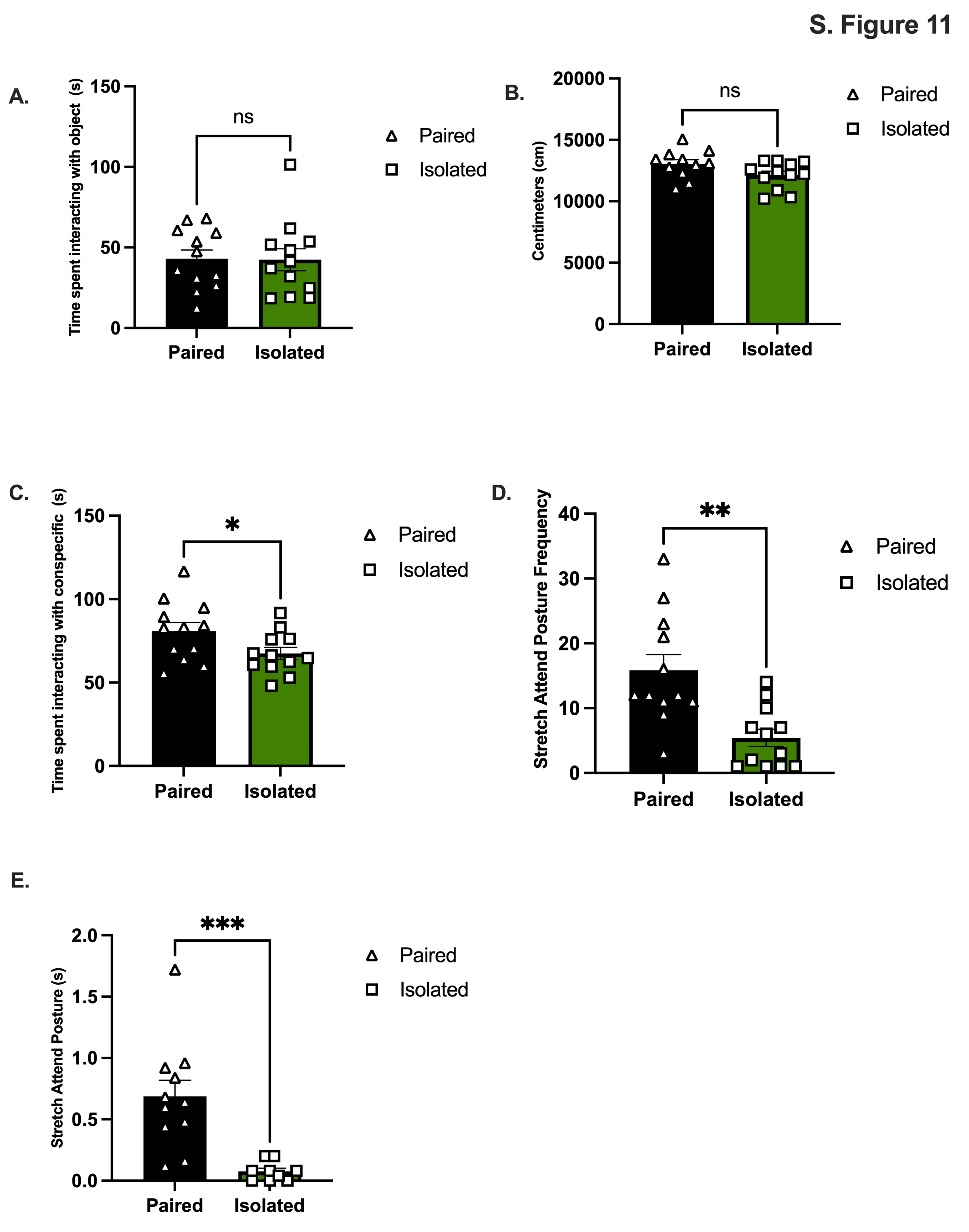
**S. Fig. 11.** Social Y Maze. Isolated have decreased sociability compared to Paired counterparts. **(A)** Time spent with the object. **(B)** the distance traveled in centimeters. **(C)** Time spent interacting with the age and sex matched conspecific. **(D)** Number of times the rats stretch attended. **(E)** Stretch attend duration in seconds. Data presented with SEM. * p < 0.05, ** p < 0.01, *** p < 0.001, **** p <0.0001; Student t- tests for parametric and Mann-Whitney for nonparametric.


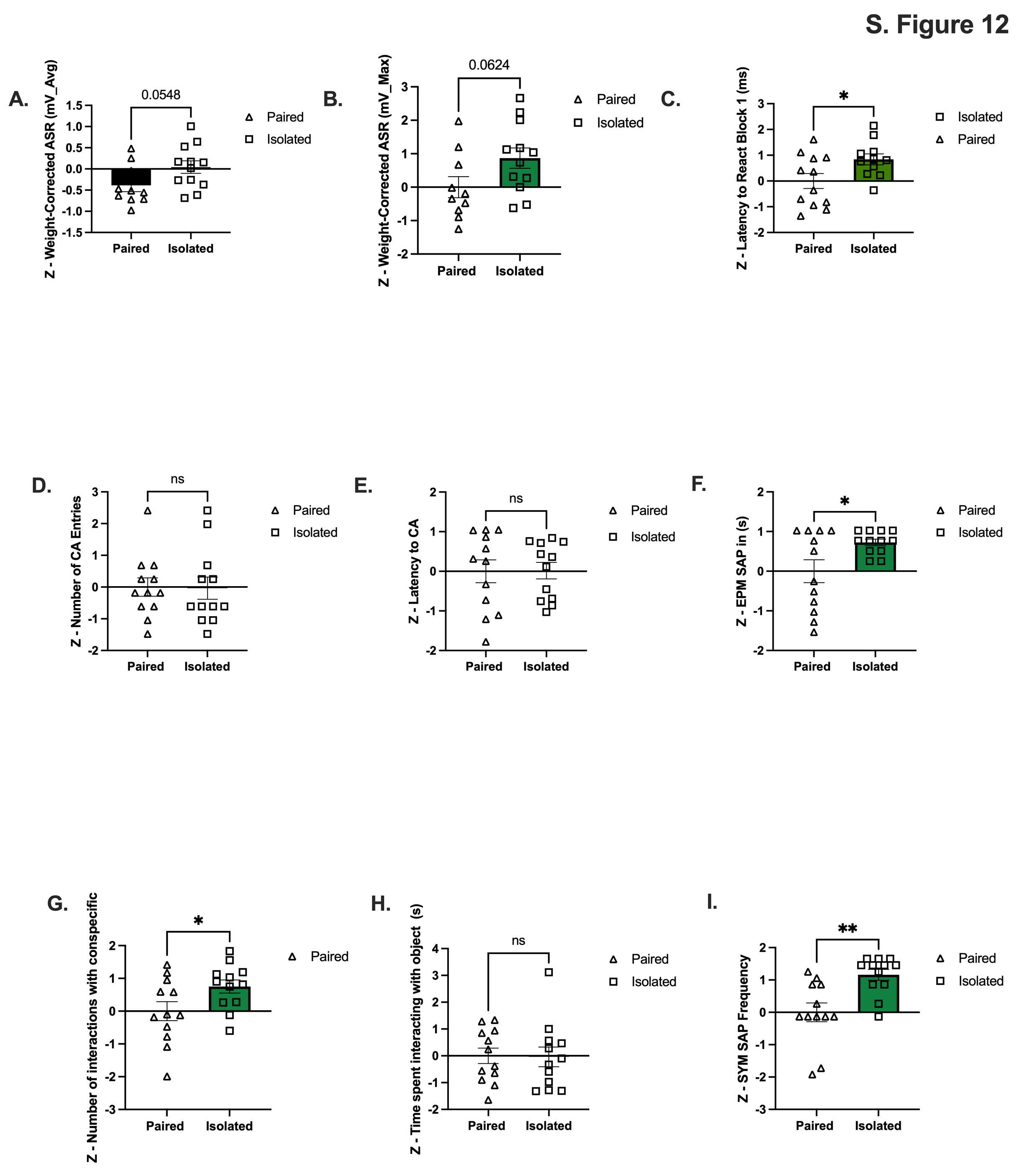
**S. Fig. 12.** Metrics used to calculate emotionality. Metrics with the highest contribution to variation based off PCA were chosen for emotionality scoring. Each metric was converted to a z-score prior to being averaged. See main figure 4. **(A-C)** ASR metrics include weight corrected average and maximum amplitude, and latency to react during the first block (10 trials). **(D-F)** EPM metrics include the number CA entries, latency to enter the CA, and duration of stretch attend postures. CA = closed arm. **(G-I)** SYM metrics include the number of interactions with the conspecific, time spent with object and stretch attend frequency. Data presented with SEM. * p < 0.05, ** p < 0.01, *** p < 0.001, **** p <0.0001; Student t- tests.

**S. Fig. 13.** Generating a social isolation-induced binge-like eating model. Lewis rats underwent chronic social isolation throughout adolescence prior to receiving WD in adulthood, n=15-16 per group. Data presented with SEM* p < 0.05, Two-Way ANOVA and Tukey HSD post hoc.


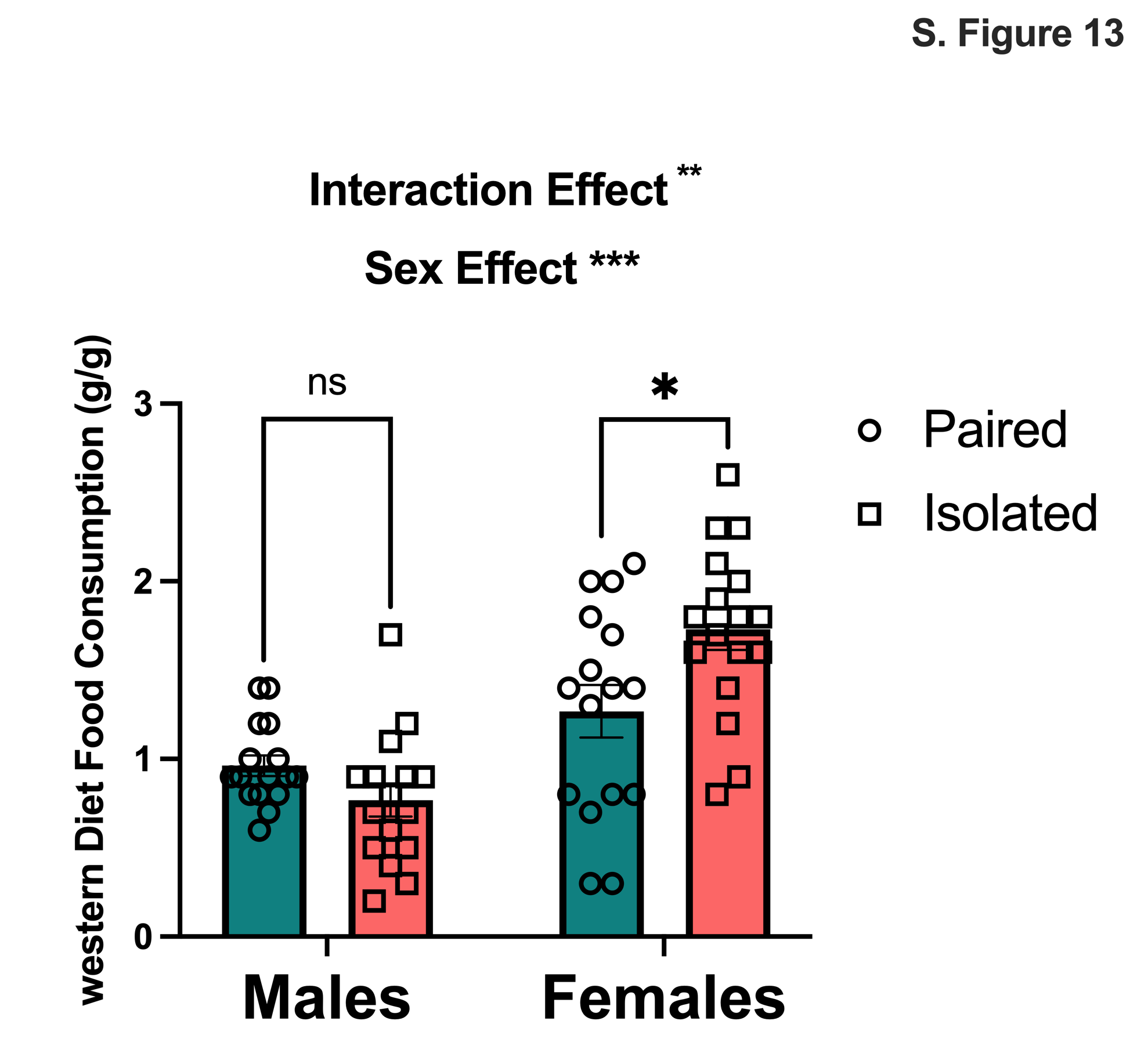


**S. Fig. 14.** Estrus cycle hormones. **(A-C)** There were no detectable differences in estrus cycle hormone levels in plasma, n=11-12. **(D)** PCA analysis with the estrus hormones further confirms overlap between groups. Data presented with SEM. Student t- tests.


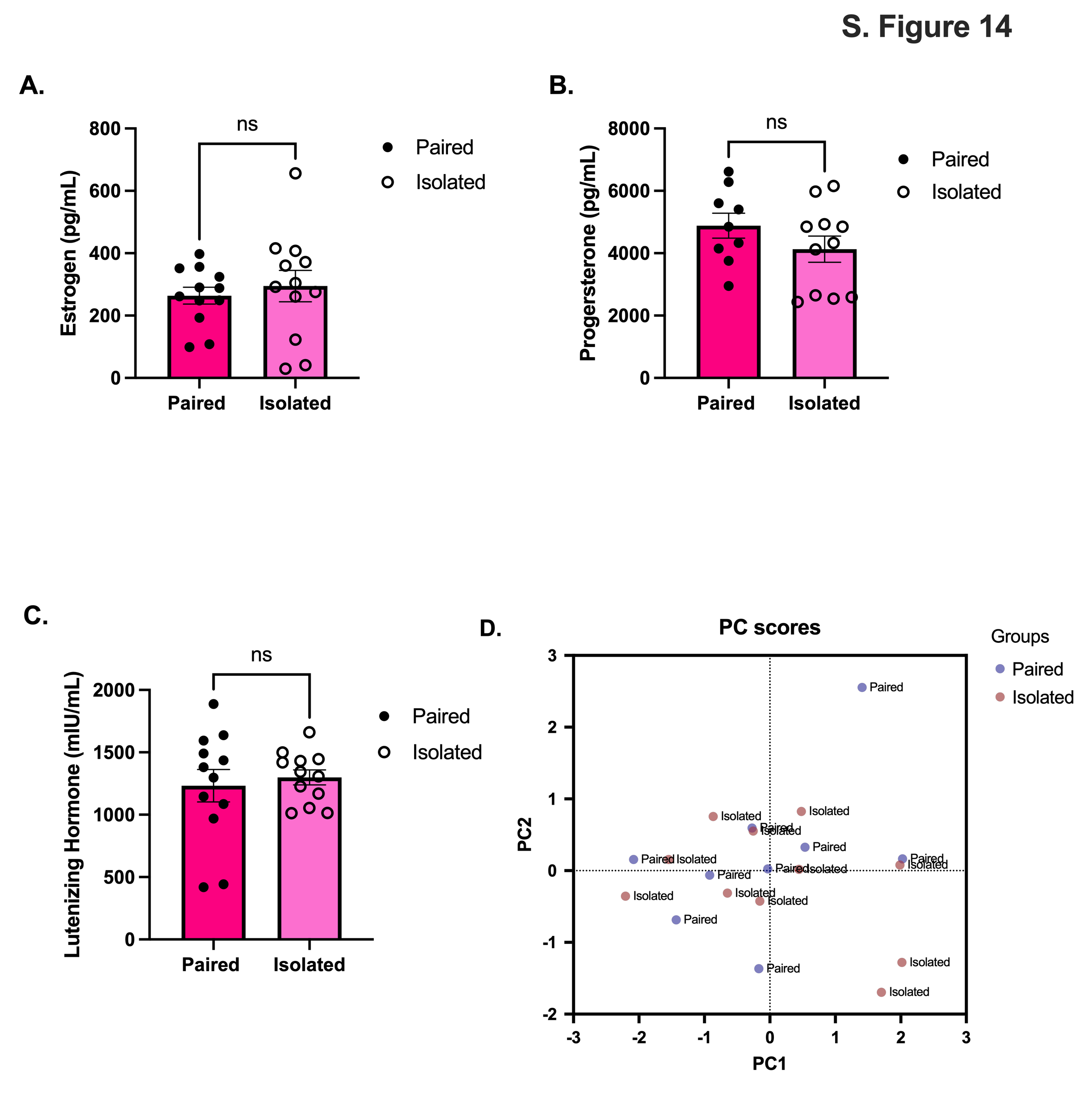


**S. Fig. 15.** Naturalistic behaviors correlate with estrus cycle hormones. **(A)** Estrogen and **(B)** Progesterone were correlated with Rearing Unsupported. **(C)** Progesterone was correlated with plasma levels of corticosterone. When separating Paired and Isolated groups, **(D)** only estrogen levels in the Paired animals remained correlated with Rearing Unsupported. **(E)** Isolated rat’s progesterone correlated with Rearing Unsupported. * p < 0.05, ** p < 0.01; Pearson Correlations conducted for each comparison. p and r values are presented.


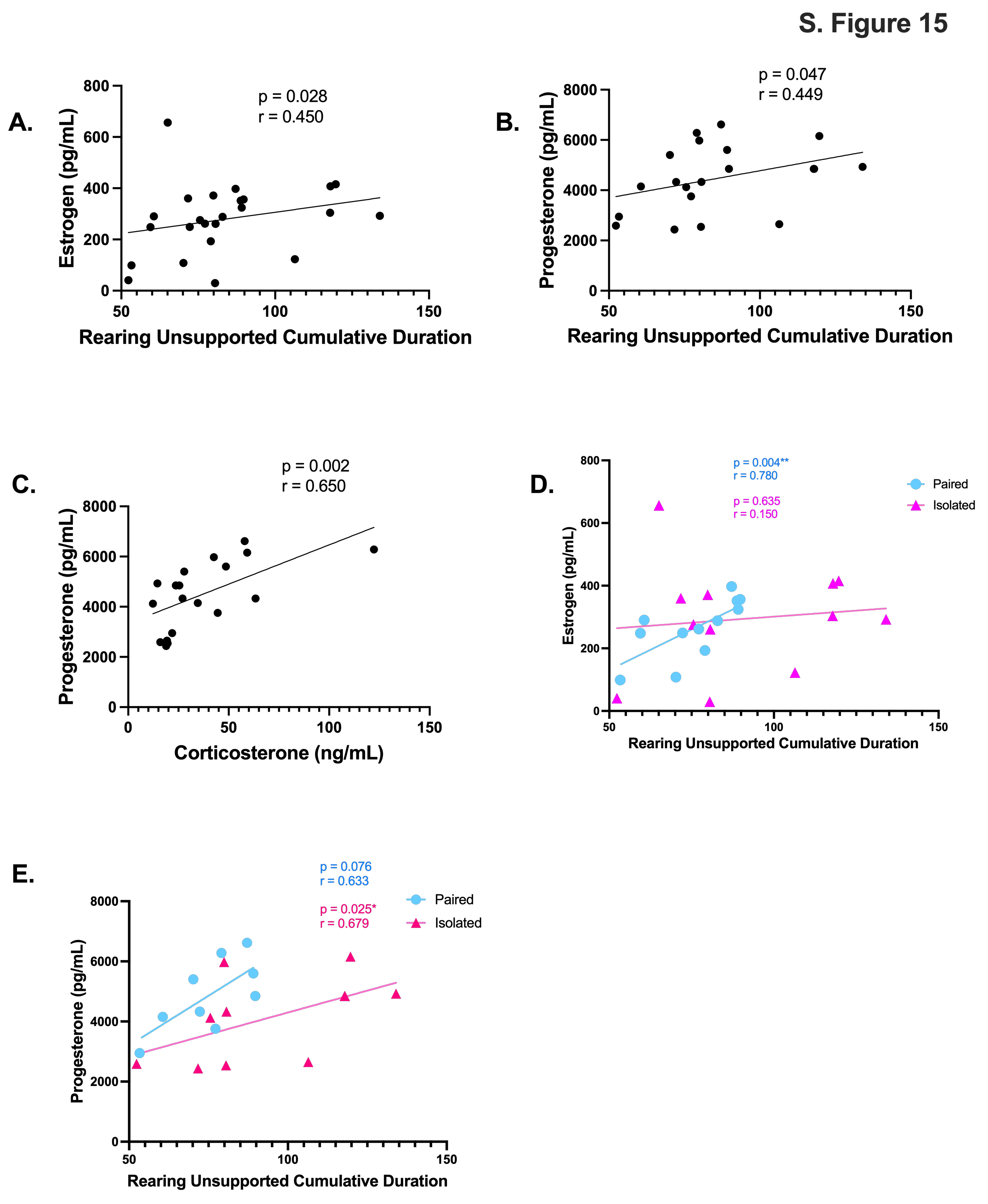


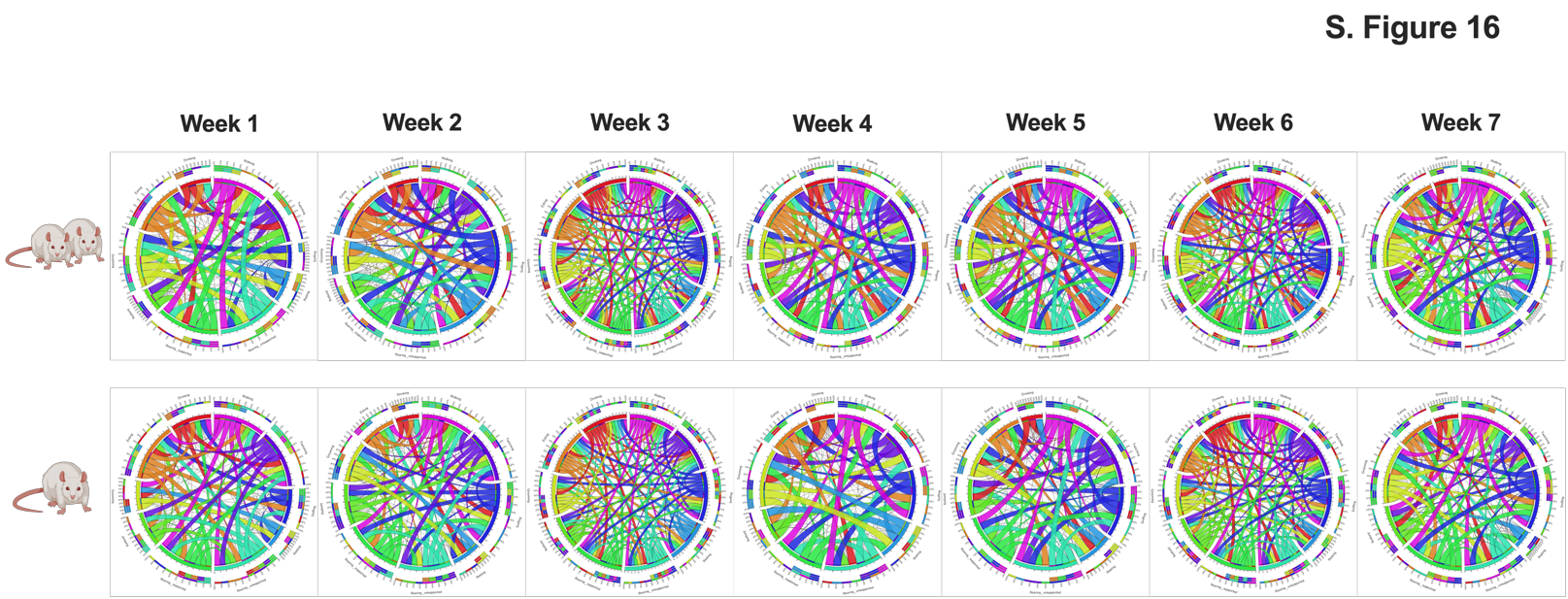
**S. Fig. 16.** Organism-level behavioral associations throughout adolescence. Connectograms display the change in behavioral associations for each behavior throughout the study. Paired animals are displayed on the top row, Isolated on the bottom. Thicker ribbons suggest strong associations.

**1.**

**10**

**9.**

**8.**

**7.**

**6.**

**5.**

**4.**

**3.**

**2.**

1. Walking
2. Twitching
3. Sniffing
4. Resting
5. Rearing Unsupported
6. Rearing Supported
7. Jumping
8. Grooming
9. Eating
10. Drinking

| **Behavior** | **Pairwise Comparisons** |  |
| --- | --- | --- |
| Adolescent Phenotypic Z Scores | t=3.218, df=22 p=0.0040 |  |
| Nonsignificant Behaviors | t=0.7506, df=22 p=0.4608 |  |
| Significant Behaviors | t=4.848, df=22 p<0.0001 |  |
|  | **2WAY ANOVA and Tukey HSD** |  |
| **Phenotypic Scores - Paired vs Isolated** |  | **Week** |
| Week 1 | p=0.9865 | F (3.064, 63.33) = 48.97 p<0.0001 |
| Week 2 | p=0.0784 |  |
| Week 3 | p=0.1764 | **Stress** |
| Week 4 | p=0.0041 | F (1, 22) = 15.91 p =0.0006 |
| Week 5 | p=0.0149 |  |
| Week 6 | p=0.0129 | **Week x Stress** |
| Week 7 | p=0.3726 | F (6, 124) = 5.388 p <0.0001 |

**2. Supplementary Tables**

**S. Table 1. Phenotypic Z-Scores**

**S. Table 2. PC1 and PC2 Scores**

| **Week** | **Pairwise Comparisons** |
| --- | --- |
| **PC 1 Scores** |  |
| Week 1 | t=0.3988, df=22, 0.6939 |
| Week 2 | t=2.312, df=22, 0.0305 |
| Week 3 | t=1.233, df=22, 0.2307 |
| Week 4 | t=2.886, df=22, 0.0086 |
| Week 5 | t=2.145, df=22, 0.0433 |
| Week 6 | t=0.4405, df=22, 0.6639 |
| Week 7 | t=0.1946, df=22, 0.8475 |
| **PC 2 Scores** |  |
| Week 1 | t=4.497, df=22, 0.0002 |
| Week 2 | t=1.022, df=22, 0.3180 |
| Week 3 | t=2.394, df=22, 0.0256 |
| Week 4 | t=0.4375, df=22, 0.6660 |
| Week 5 | t=1.017, df=22, 0.3201 |
| Week 6 | t=3.379, df=22, 0.0027 |
| Week 7 | t=0.5471, df=22, 0.5898 |

**S. Table 3. Weekly Behaviors.**

| **Behaviors** | **Weekly** | **Stress** | **Interaction** |
| --- | --- | --- | --- |
| Eating | F (6, 146) = 2.865 p=0.0114 | F (1, 146) = 1.208 p=0.2736 | F (6, 146) = 0.4740 p=0.8268 |
| Drinking | F (6, 146) = 4.279 p=0.0005 | F (1, 146) = 0.6477 p=0.4223 | F (6, 146) = 0.1239 p=0.9933 |
| Grooming | F (6, 146) = 21.62 p<0.0001 | F (1, 146) = 0.0002278 p=0.9880 | F (6, 146) = 0.2157 p=0.9713 |
| Jumping | F (6, 146) = 19.99 p<0.0001 | F (1, 146) = 65.83 p<0.0001 | F (6, 146) = 3.169 p=0.0059 |
| Rearing Supported | F (6, 146) = 32.67 p<0.0001 | F (1, 146) = 0.2944 p=0.5882 | F (6, 146) = 1.962 p=0.0747 |
| Rearing Unsupported | F (6, 146) = 5.017 p=0.0001 | F (1, 146) = 6.797 p=0.0101 | F (6, 146) = 1.020 p=0.4148 |
| Resting | F (6, 146) = 0.8958 p=0.4998 | F (1, 146) = 1.694 p=0.1951 | F (6, 146) = 0.3960 p=0.8807 |
| Sniffing | F (6, 146) = 15.29 p<0.0001 | F (1, 146) = 18.90 p<0.0001 | F (6, 146) = 1.157 p=0.3328 |
| Twitching | F (6, 146) = 7.920 p<0.0001 | F (1, 146) = 68.16 p<0.0001 | F (6, 146) = 1.623 p=0.1448 |
| Walking | F (6, 146) = 19.50 p<0.0001 | F (1, 146) = 0.01932 p=0.8897 | F (6, 146) = 1.379 p=0.2267 |

**S. Table 4. Hourly Behaviors.**

| Behaviors | Interactions | Hours | Stress |
| --- | --- | --- | --- |
| Week 1 Drinking | F (12, 258) = 0.5402 P=0.8873 | F (12, 258) = 5.384 P<0.0001 | F (1, 258) = 1.431 P=0.2326 |
| Week 2 Drinking | F (12, 286) = 2.172 P=0.0131 | F (12, 286) = 7.175 P<0.0001 | F (1, 286) = 0.08270 P=0.7739 |
| Week 3 Drinking | F (12, 234) = 0.5940 P=0.8462 | F (12, 234) = 3.876 P<0.0001 | F (1, 234) = 0.4226 P=0.5163 |
| Week 4 Drinking | F (12, 286) = 0.8998 P=0.5476 | F (12, 286) = 6.136 P<0.0001 | F (1, 286) = 0.09116 P=0.7629 |
| Week 5 Drinking | F (12, 286) = 0.9431 P=0.5041 | F (12, 286) = 8.122 P<0.0001 | F (1, 286) = 0.3793 P=0.5385 |
| Week 6 Drinking | F (12, 234) = 0.5588 P=0.8736 | F (12, 234) = 3.253 P=0.0002 | F (1, 234) = 0.4332 P=0.5111 |
| Week 7 Drinking | F (12, 286) = 1.009 P=0.4402 | F (12, 286) = 5.508 P<0.0001 | F (1, 286) = 0.01138 P=0.9151 |
| Week 1 Eating | F (12, 258) = 0.9126 P=0.5349 | F (12, 258) = 5.312 P<0.0001 | F (1, 258) = 0.1521 P=0.6969 |
| Week 2 Eating | F (12, 286) = 1.463 P=0.1376 | F (12, 286) = 5.654 P<0.0001 | F (1, 286) = 0.3940 P=0.5307 |
| Week 3 Eating | F (12, 234) = 0.7173 P=0.7340 | F (12, 234) = 3.732 P<0.0001 | F (1, 234) = 0.8291 P=0.3635 |
| Week 4 Eating | F (12, 286) = 0.4968 P=0.9160 | F (12, 286) = 8.109 P<0.0001 | F (1, 286) = 0.1769 P=0.6744 |
| Week 5 Eating | F (12, 286) = 0.2643 P=0.9939 | F (12, 286) = 8.681 P<0.0001 | F (1, 286) = 2.723 P=0.1000 |
| Week 6 Eating | F (12, 234) = 0.1475 P=0.9997 | F (12, 234) = 5.323 P<0.0001 | F (1, 234) = 0.4774 P=0.4903 |
| Week 7 Eating | F (12, 286) = 1.454 P=0.1411 | F (12, 286) = 11.07 P<0.0001 | F (1, 286) = 1.688 P=0.1949 |
| Week 1 Grooming | F (12, 258) = 1.239 P=0.2564 | F (12, 258) = 7.648 P<0.0001 | F (1, 258) = 0.4605 P=0.4980 |
| Week 2 Grooming | F (12, 286) = 1.219 P=0.2693 | F (12, 286) = 4.557 P<0.0001 | F (1, 286) = 1.556 P=0.2133 |
| Week 3 Grooming | F (12, 234) = 1.002 P=0.4475 | F (12, 234) = 3.430 P=0.0001 | F (1, 234) = 0.8444 P=0.3591 |
| Week 4 Grooming | F (12, 286) = 0.5908 P=0.8492 | F (12, 286) = 4.950 P<0.0001 | F (1, 286) = 0.05130 P=0.8210 |
| Week 5 Grooming | F (12, 286) = 0.7158 P=0.7358 | F (12, 286) = 4.987 P<0.0001 | F (1, 286) = 0.5084 P=0.4764 |
| Week 6 Grooming | F (12, 234) = 0.4618 P=0.9352 | F (12, 234) = 3.078 P=0.0005 | F (1, 234) = 2.267 P=0.1335 |
| Week 7 Grooming | F (12, 286) = 1.045 P=0.4079 | F (12, 286) = 5.900 P<0.0001 | F (1, 286) = 0.4358 P=0.5097 |
| Week 1 Jumping | F (12, 258) = 1.284 P=0.2279 | F (12, 258) = 2.456 P=0.0048 | F (1, 258) = 17.35 P<0.0001 |
| Week 2 Jumping | F (12, 286) = 2.730 P=0.0016 | F (12, 286) = 3.863 P<0.0001 | F (1, 286) = 53.21 P<0.0001 |
| Week 3 Jumping | F (12, 234) = 0.8427 P=0.6063 | F (12, 234) = 1.251 P=0.2490 | F (1, 234) = 35.38 P<0.0001 |
| Week 4 Jumping | F (12, 286) = 1.235 P=0.2584 | F (12, 286) = 5.000 P<0.0001 | F (1, 286) = 48.03 P<0.0001 |
| Week 5 Jumping | F (12, 286) = 1.879 P=0.0366 | F (12, 286) = 9.750 P<0.0001 | F (1, 286) = 50.16 P<0.0001 |
| Week 6 Jumping | F (12, 234) = 1.653 P=0.0784 | F (12, 234) = 6.724 P<0.0001 | F (1, 234) = 37.69 P<0.0001 |
| Week 7 Jumping | F (12, 286) = 2.407 P=0.0055 | F (12, 286) = 11.86 P<0.0001 | F (1, 286) = 18.93 P<0.0001 |
| Week 1 Rearing | F (12, 258) = 0.3860 P=0.9678 | F (12, 258) = 13.40 P<0.0001 | F (1, 258) = 0.2080 P=0.6487 |
| Week 2 Rearing | F (12, 286) = 0.9270 P=0.5201 | F (12, 286) = 10.40 P<0.0001 | F (1, 286) = 2.423 P=0.1207 |
| Week 3 Rearing | F (12, 234) = 1.306 P=0.2155 | F (12, 234) = 6.049 P<0.0001 | F (1, 234) = 11.03 P=0.0010 |
| Week 4 Rearing | F (12, 286) = 0.6445 P=0.8033 | F (12, 286) = 14.74 P<0.0001 | F (1, 286) = 3.206 P=0.0744 |
| Week 5 Rearing | F (12, 286) = 0.6247 P=0.8208 | F (12, 286) = 8.258 P<0.0001 | F (1, 286) = 2.376 P=0.1243 |
| Week 6 Rearing | F (12, 234) = 0.5550 P=0.8764 | F (12, 234) = 9.158 P<0.0001 | F (1, 234) = 7.748 P=0.0058 |
| Week 7 Rearing | F (12, 286) = 1.016 P=0.4342 | F (12, 286) = 7.233 P<0.0001 | F (1, 286) = 2.096 P=0.1488 |
| Week 1 Rearing Unsp | F (12, 258) = 1.393 P=0.1692 | F (12, 258) = 4.468 P<0.0001 | F (1, 258) = 0.2946 P=0.5878 |
| Week 2 Rearing Unsp | F (12, 286) = 1.679 P=0.0707 | F (12, 286) = 5.630 P<0.0001 | F (1, 286) = 3.452 P=0.0642 |
| Week 3 Rearing Unsp | F (12, 234) = 1.078 P=0.3793 | F (12, 234) = 5.494 P<0.0001 | F (1, 234) = 14.23 P=0.0002 |
| Week 4 Rearing Unsp | F (12, 286) = 0.4985 P=0.9149 | F (12, 286) = 11.48 P<0.0001 | F (1, 286) = 2.523 P=0.1133 |
| Week 5 Rearing Unsp | F (12, 286) = 0.3993 P=0.9633 | F (12, 286) = 9.995 P<0.0001 | F (1, 286) = 6.526 P=0.0111 |
| Week 6 Rearing Unsp | F (12, 234) = 0.7520 P=0.6994 | F (12, 234) = 6.965 P<0.0001 | F (1, 234) = 0.01122 P=0.9157 |
| Week 7 Rearing Unsp | F (12, 286) = 1.049 P=0.4037 | F (12, 286) = 6.698 P<0.0001 | F (1, 286) = 0.03316 P=0.8556 |
| Week 1 Resting | F (12, 258) = 1.515 P=0.1186 | F (12, 258) = 2.558 P=0.0033 | F (1, 258) = 1.560 P=0.2128 |
| Week 2 Resting | F (12, 286) = 0.6619 P=0.7874 | F (12, 286) = 1.473 P=0.1337 | F (1, 286) = 5.572 P=0.0189 |
| Week 3 Resting | F (12, 234) = 0.4584 P=0.9369 | F (12, 234) = 1.058 P=0.3966 | F (1, 234) = 0.1583 P=0.6911 |
| Week 4 Resting | F (12, 286) = 0.4019 P=0.9623 | F (12, 286) = 3.018 P=0.0005 | F (1, 286) = 0.03398 P=0.8539 |
| Week 5 Resting | F (12, 286) = 0.5771 P=0.8602 | F (12, 286) = 2.865 P=0.0010 | F (1, 286) = 0.006831 P=0.9342 |
| Week 6 Resting | F (12, 234) = 1.072 P=0.3847 | F (12, 234) = 2.194 P=0.0127 | F (1, 234) = 5.160 P=0.0240 |
| Week 7 Resting | F (12, 286) = 1.235 P=0.2585 | F (12, 286) = 3.105 P=0.0004 | F (1, 286) = 0.07661 P=0.7821 |
| Week 1 Sniffing | F (12, 258) = 0.5875 P=0.8517 | F (12, 258) = 6.344 P<0.0001 | F (1, 258) = 0.04524 P=0.8317 |
| Week 2 Sniffing | F (12, 286) = 1.791 P=0.0492 | F (12, 286) = 6.370 P<0.0001 | F (1, 286) = 14.61 P=0.0002 |
| Week 3 Sniffing | F (12, 234) = 1.555 P=0.1058 | F (12, 234) = 4.052 P<0.0001 | F (1, 234) = 34.44 P<0.0001 |
| Week 4 Sniffing | F (12, 286) = 0.5400 P=0.8877 | F (12, 286) = 9.700 P<0.0001 | F (1, 286) = 16.44 P<0.0001 |
| Week 5 Sniffing | F (12, 286) = 0.9258 P=0.5214 | F (12, 286) = 7.295 P<0.0001 | F (1, 286) = 16.82 P<0.0001 |
| Week 6 Sniffing | F (12, 234) = 0.5672 P=0.8673 | F (12, 234) = 6.535 P<0.0001 | F (1, 234) = 1.640 P=0.2017 |
| Week 7 Sniffing | F (12, 286) = 1.158 P=0.3133 | F (12, 286) = 7.985 P<0.0001 | F (1, 286) = 1.928 P=0.1661 |
| Week 1 Twitching | F (12, 257) = 1.961 P=0.0282 | F (12, 257) = 10.02 P<0.0001 | F (1, 257) = 23.27 P<0.0001 |
| Week 2 Twitching | F (12, 286) = 2.071 P=0.0188 | F (12, 286) = 5.490 P<0.0001 | F (1, 286) = 25.74 P<0.0001 |
| Week 3 Twitching | F (12, 234) = 1.296 P=0.2216 | F (12, 234) = 3.491 P<0.0001 | F (1, 234) = 40.47 P<0.0001 |
| Week 4 Twitching | F (12, 286) = 1.042 P=0.4101 | F (12, 286) = 6.346 P<0.0001 | F (1, 286) = 19.56 P<0.0001 |
| Week 5 Twitching | F (12, 286) = 1.039 P=0.4132 | F (12, 286) = 7.242 P<0.0001 | F (1, 286) = 37.49 P<0.0001 |
| Week 6 Twitching | F (12, 234) = 1.316 P=0.2099 | F (12, 234) = 9.735 P<0.0001 | F (1, 234) = 10.84 P=0.0011 |
| Week 7 Twitching | F (12, 286) = 1.651 P=0.0773 | F (12, 286) = 8.310 P<0.0001 | F (1, 286) = 3.481 P=0.0631 |
| Week 1 Walking | F (12, 258) = 0.4536 P=0.9396 | F (12, 258) = 7.709 P<0.0001 | F (1, 258) = 3.666 P=0.0566 |
| Week 2 Walking | F (12, 286) = 1.677 P=0.0712 | F (12, 286) = 10.07 P<0.0001 | F (1, 286) = 2.356 P=0.1259 |
| Week 3 Walking | F (12, 234) = 1.148 P=0.3224 | F (12, 234) = 4.008 P<0.0001 | F (1, 234) = 9.146 P=0.0028 |
| Week 4 Walking | F (12, 286) = 0.6495 P=0.7988 | F (12, 286) = 12.47 P<0.0001 | F (1, 286) = 1.620 P=0.2041 |
| Week 5 Walking | F (12, 286) = 0.5671 P=0.8679 | F (12, 286) = 6.471 P<0.0001 | F (1, 286) = 1.577 P=0.2103 |
| Week 6 Walking | F (12, 234) = 0.5562 P=0.8755 | F (12, 234) = 8.588 P<0.0001 | F (1, 234) = 10.05 P=0.0017 |
| Week 7 Walking | F (12, 286) = 0.9182 P=0.5290 | F (12, 286) = 6.178 P<0.0001 | F (1, 286) = 1.479 P=0.2249 |

**S. Table 5. Residual Avoidance. Two-Way ANOVA.**

| **Behavior** | **Weeks** | **Stress** | **Interaction** |
| --- | --- | --- | --- |
| Shelter Zone | F (6, 141) = 1.511 p=0.1786 | F (1, 141) = 2.283 p=0.1330 | F (6, 141) = 1.511 p=0.1786 |
| Food Zone | F (6, 141) = 0.7140 p=0.6389 | F (1, 146) = 1.461 p=0.0470 | F (6, 141) = 0.7140 p=0.6389 |
| Food Zone during spotlight | F (6, 141) = 0.9163  p=0.4852 | F (1, 141) = 7.545  p=0.0068 | F (6, 141) = 0.6354  p=0.7017 |

**S. Table 6. Acoustic Startle Reactivity**

| **Behavior** | **Pairwise Comparison** |
| --- | --- |
| mV_Max | t=1.974, df=20 p=0.0624 |
| T_Max | t=0.9167, df=21 p=0.3697 |
| mV_AVG | t=2.040, df=20 p=0.0548 |
| **mV_Max** |  |
| Block 1 | t=1.777, df=20 p=0.0908 |
| Block 2 | t=0.9544, df=21 p=0.3508 |
| Block 3 | t=1.484, df=20, p-0.1535 |
| **mV_AVG** |  |
| Block 1 | t=1.527, df=20 p= 0.1424 |
| Block 2 | t=0.9202, df=21 p=0.3679 |
| Block 3 | t=1.370, df=21 p=0.1853 |

**S. Table 7. Elevated Plus Maze**

| **Behavior** | **Pairwise Comparison** |
| --- | --- |
| Anxiety Index | t=1.070, df=22 p=0.2961 |
| **CA** |  |
| Time | t=1.174, df=21 p=0.2535 |
| Frequency | t=1.213, df=22 p=0.2380 |
| **OA** |  |
| Time | t=1.174, df=21 p=0.2535 |
| Frequency | t=1.213, df=22 p=0.2380 |
| **Body Elongation Stretch** |  |
| Frequency | t=2.224, df=22 p=0.0367 |
| Distanced moved (cm) | t=0.7794, df=22 p=0.4441 |

**S. Table 8. Social Y Maze**

| **Behavior** | **Pairwise Comparison** |
| --- | --- |
| **Object** |  |
| Frequency | Sum of Ranks (127, 104), p=0.6873 |
| CD | t=0.08243, df=22 p=0.9350 |
| **Conspecific** |  |
| Frequency | t=1.392, df=22 p=0.1779 |
| CD | t=2.143, df=22 p=0.0434 |
| **Body Elongation Stretch** |  |
| Frequency | t=3.467, df=22 p=0.0022 |
| CD | Sum of Ranks (161, 49), p<0.0001 |
| **Distance Traveled** | t=1.934, df=21 p=0.0667 |

**S. Table 9. Emotionality**

| **Behavior** | **Student t** |
| --- | --- |
| **SYM Z Norm** |  |
| Z Norm Conspecific Duration | t=2.143, df=22 p=0.0434 |
| Z Norm SAP Frequency | t=3.467, df=22 p=0.0022 |
| Z Norm Object Duration | t=0.08243, df=22 p=0.9350 |
| **EPM Z Norm** |  |
| Z Norm CA Frequency | t=0.07935, df=22 p=0.9375 |
| Z Norm Latency to CA | t=0.04545, df=22 p=0.9642 |
| Z Norm SAP Duration | t=2.415, df=22 p=0.0245 |
| **ASR** |  |
| Z Norm mV_AVG | t=2.040, df=20 p=0.0548 |
| Z Norm T_Max Block 1 | t=2.314, df=21 p=0.0309 |
| Z Norm mV_Max | t=1.974, df=20 p=0.0624 |
|  |  |
| **Emotionality** | t=4.017, df=22 p=0.0006 |
| SYM Z Avg | t=2.139, df=20 p=0.0449 |
| EPM Z Avg | t=1.008, df=22 p=0.3243 |
| ASR Z Avg | t=3.693, df=22 p=0.0013 |

**S. Table 10. Divergent Sex Effects**

|  | **Two-Way ANOVA and Tukey HSD Post Hoc Comparisons** |
| --- | --- |
| Interaction | F (1, 61) = 9.032 p=0.0038 |
| Sex | F (1, 61) = 33.85 p<0.0001 |
| Stress | F (1, 61) = 1.503 p=0.2250 |
| Males Paired v Males Isolated | p=0.5985 |
| Females Paired v Females Isolated | p=0.0191 |

**S. Table 11. Binge-Like Eating and Correlations**

| BED Hyperphagic | t=3.588, df=9 p=0.005 | Pairwise comparisons |
| --- | --- | --- |
| Twitching and WD consumption | r= 0.5552, p=0.0049, n=24 | Pearson correlation |
| Jumping and WD consumption | r= 0.5150, p=0.0102, n=24 | Pearson correlation |
| Hyperphagic and hypophagic twitching comparisons | Interaction: F (1,20)=3.063, p=0.0954  Binge-like Effect: F (1,20) =5.032, p=0.0364  Stress Effect: F (1,20) =35.28, p<0001   \| Hyperphagic:Paired vs. Hyperphagic:Isolated, p=0.0002 \| \| --- \| \| Hyperphagic:Paired vs. Hypophagic:Paired, p=0.9847 \| \| Hyperphagic:Paired vs. Hypophagic:Isolated, p=0573 \| \| Hyperphagic:Isolated vs. Hypophagic:Paired, p=0.0001 \| \| Hyperphagic:Isolated vs. Hypophagic:Isolated, p=0.0495 \| \| Hypophagic:Paired vs. Hypophagic:Isolated, p=0.0269 \| | Two-Way ANOVA with Tukey HSD post hoc |
|  | Paired | Isolated |
| Jumping and BED Paired Isolated Separate | r= 0.047, p=0.884 | r= 0.570, p=0.053 |
| Twitching and BED Paired Isolated Separate | r= 0.265, p=0.405 | r= 0.657, p=0.020 |

**S. Table 12. Endocrine**

|  | Pairwise Comparisons |
| --- | --- |
| CORT | t=4.006, df=20 p=0.0007 |
| Estrogen | t=0.5395, df=22 p=0.5949 |
| Progesterone | t=1.280, df=18 p=0.2170 |
| LH | t=0.4650, df=22 p=0.6465 |
|  | Spearman Correlation |
| Adolescent Shifted Behavioral z-score and CORT | r=-0.428, p=0.042 |
| Emotionality and CORT | r=-0.418, p=0.0421 |
|  | Pearson Correlation |
| Jumping and Cort | r=-0.404, p=0.050 |
| Twitching and CORT | r=-0.463, p=0.023 |
